# *Aspergillus sydowii*: Genome Analysis and Characterization of Two Heterologous Expressed, Non-redundant Xylanases

**DOI:** 10.3389/fmicb.2020.573482

**Published:** 2020-09-18

**Authors:** Sophie C. Brandt, Bernhard Ellinger, Thuat van Nguyen, Sönke Harder, Hartmut Schlüter, Richard L. Hahnke, Martin Rühl, Wilhelm Schäfer, Martin Gand

**Affiliations:** ^1^Department of Molecular Phytopathology, University of Hamburg, Hamburg, Germany; ^2^Department ScreeningPort, Fraunhofer Institute for Molecular Biology and Applied Ecology IME, Hamburg, Germany; ^3^Mass Spectrometric Proteomics Group, Institute of Clinical Chemistry and Laboratory Medicine, University Medical Center Hamburg-Eppendorf, Hamburg, Germany; ^4^Department of Microorganisms, Leibniz Institute DSMZ – German Collection of Microorganisms and Cell Cultures, Braunschweig, Germany; ^5^Institute of Food Chemistry and Food Biotechnology, Justus Liebig University Giessen, Giessen, Germany

**Keywords:** *Aspergillus sydowii*, xylanase, mass spectrometry, heterologous expression, glycoside hydrolases 10 and 11, biocatalysis, site directed mutagenesis

## Abstract

A prerequisite for the transition toward a biobased economy is the identification and development of efficient enzymes for the usage of renewable resources as raw material. Therefore, different xylanolytic enzymes are important for efficient enzymatic hydrolysis of xylan-heteropolymers. A powerful tool to overcome the limited enzymatic toolbox lies in exhausting the potential of unexplored habitats. By screening a Vietnamese fungal culture collection of 295 undiscovered fungal isolates, 12 highly active xylan degraders were identified. One of the best xylanase producing strains proved to be an *Aspergillus sydowii* strain from shrimp shell (Fsh102), showing a specific activity of 0.6 U/mg. Illumina dye sequencing was used to identify our Fsh102 strain and determine differences to the *A. sydowii* CBS 593.65 reference strain. With activity based in-gel zymography and subsequent mass spectrometric identification, three potential proteins responsible for xylan degradation were identified. Two of these proteins were cloned from the cDNA and, furthermore, expressed heterologously in *Escherichia coli* and characterized. Both xylanases, were entirely different from each other, including glycoside hydrolases (GH) families, folds, substrate specificity, and inhibition patterns. The specific enzyme activity applying 0.1% birch xylan of both purified enzymes were determined with 181.1 ± 37.8 or 121.5 ± 10.9 U/mg for xylanase I and xylanase II, respectively. Xylanase I belongs to the GH11 family, while xylanase II is member of the GH10 family. Both enzymes showed typical endo-xylanase activity, the main products of xylanase I are xylobiose, xylotriose, and xylohexose, while xylobiose, xylotriose, and xylopentose are formed by xylanase II. Additionally, xylanase II showed remarkable activity toward xylotriose. Xylanase I is stable when stored up to 30°C and pH value of 9, while xylanase II started to lose significant activity stored at pH 9 after exceeding 3 days of storage. Xylanase II displayed about 40% activity when stored at 50°C for 24 h. The enzymes are tolerant toward mesophilic temperatures, while acting in a broad pH range. With site directed mutagenesis, the active site residues in both enzymes were confirmed. The presented activity and stability justify the classification of both xylanases as highly interesting for further development.

## Introduction

Due to the rising population and the increasing global wealth, the demand for energy is constantly increasing (Sorrell, 2015). As a consequence, the petrochemical reserves are decreasing (Miller and Sorrell, 2014). As a result of the global climate change and the accelerated consumption of fossil fuels (Sommer, 2016), a general scientific and political rethink had taken place in the last decade. A transition to biobased products is, therefore, necessary [National Research Council (US) Committee on Biobased Industrial Products, 2000]. Renewable fuels (Sims et al., 2010) and other chemicals (Straathof, 2014) can be generated from plant materials. As a result of these efforts, the first generation of biofuels, which are produced directly from food crops, can substitute fossil fuels (Mohr and Raman, 2013). This first generation suffers in the limitations of the competition between food or feed and the biomass for the biofuels (Valentine et al., 2012). To overcome these limitations, the second generation biofuels based on plant waste material as a source for bioethanol were developed (Saini et al., 2015) and, at present, second-generation biofuels are still under development. The production of lignocellulosic plant waste material is estimated to be currently about 5 billion metric tons per year (Global Partnership on Waste Management, 2011). This amount of material has a stored energy equivalent to 1.2 billion metric tons of oil (Global Partnership on Waste Management, 2011). These numbers highlight that plant waste biomass is an important resource for renewable energy. The hydrolysis of biomass to monosaccharides is still challenging due to the complex plant cell-wall structure, being a mixed composition of cellulose, hemicellulose, and lignin (Gilbert, 2010; Agbor et al., 2011; Singhvi et al., 2014). Hemicellulose is the second most abundant polysaccharide in nature, in land plants the amount of xylan, the major constituent of hemicellulose, is 20–40% of the total dry weight (Bajpai, 2014). These hemicellulose macromolecules are loosely structured, consisting of polymers of pentoses (xylose and arabinose) and hexoses (mostly mannose) in varying ratios (Holtzapple, 2003). Xylan, the main component, consists of a linear backbone of β-d-xylopyranosyl sugars chemically linked by β-(1,4) glycosidic bonds. This backbone is substituted with 4-O-methyl-α-d-glucuronopyranosyl units, acetyl groups or α-l-arabinofuranosyl units, in different distribution (Bajpai, 2014). The complete biodegradation requires cooperation of several enzymes, namely endo-1,4-β-xylanases (EC 3.2.1.8), β-d-xylosidases (EC 3.2.1.37), α-l-arabinofuranosidases (EC 3.2.1.55), α-glucuronidases (EC 3.2.1.139), acetyl xylan esterases (EC 3.1.1.72), and ferulic acid esterases (EC 3.1.1.73; Shallom and Shoham, 2003). Sometimes exo-xylanases (EC 3.2.1.37) are completing the toolbox of hydrolytic hemicellulases. Among them, endo-1,4-β-xylanase plays an important role in the degradation of xylan by hydrolyzing the xylosyl backbone into smaller xylooligosaccharides (XOS). These enzymes belong to the GH10 and GH11 groups of glycoside hydrolases (GH; Henrissat, 1991). The GH family 10 was assigned to GH-A clan (Davies and Sinnott, 2008), because of their TIM barrel-(β/α)_8_ structure, whereas GH family 11 was allocated to GH-C clan (Davies and Sinnott, 2008), because of their β-jelly roll structure. The next step in the degradation cascade is the hydrolysis of the XOS into β-d-xylose residues by 1,4-β-xylosidases. After xylan degradation, the recalcitrant lignocellulose is more amendable to further degradation by other lignocellulosic enzymes (Chandra et al., 2007). Generally in industrial approaches, a chemical or physical pretreatment is used to reduce lignin content for an easier work flow (Chandra et al., 2007). Beside biofuels, xylanases are used for various other industrial processes such as food, feed, and pulp and paper industries (Beg et al., 2001). These current applications include prebleaching of pulp to reduce the amount of chemical bleaching (Walia et al., 2017), increasing the loaf volumes in baking (Butt et al., 2008), enhancing the weight gain of broiler chickens by incorporation of xylanase into the rye-based diet food (Bedford et al., 1991), clarifying juices of fruits and vegetables (Biely, 1985), and treating the wastewater from agricultural production (Biely, 1985).

In nature, the lignocellulosic biomass is mainly decomposed by fungi (Guerriero et al., 2016). The fungal kingdom includes over 5 million species, and it is estimated that only 5% are formally classified (Mueller and Bills, 2004; Blackwell, 2011). The Ascomycota are considered to have exceptional relevance for mankind (Bennett, 1998), in consequence of their potential to produce pharmaceuticals (Gibbs et al., 2000) and other chemicals (van der Straat et al., 2014), as well as their application in food and beverage industry (Ferreira et al., 2016). *Aspergillus* is one of the most abundant genus in that phylum (Klich, 2002). Depending on the growth media, they exhibit characteristic colors, from black, to gray or green up to white or milky. Over 250 species of the genera, *Aspergillus* are currently known. A less studied fungus of that genus is *Aspergillus sydowii*, which was first described in 1913 as *Sterigmatocystis sydowii* by Bainier and Sartory (1913). In 1926, the fungus was reclassified as *A. sydowii* (Thom and Church, 1926). The fungus is distributed all over the world and can occur in different environments, where it survives as a saprotroph (Raper and Fennell, 1965; Geiser et al., 1998; Klich, 2002). Beside its terrestrial appearance in soil, it can grow in the sea due to its salt tolerance (Hallegraeff et al., 2014). In addition, *A. sydowii* can cause human infections (Rinaldi, 1983) as well as contaminate food (Piotrowska, 2013; Biango-Daniels and Hodge, 2018). In marine ecosystems, it causes epizootic infections of sea fan corals (Geiser et al., 1998; Alker et al., 2001; Kim and Harvell, 2004).

Within this study, we aimed to investigate an *A. sydowii* strain, which originates from shrimp shell, and its endo-xylanases. This strain was part of a collection previously isolated from nine different substrates and environments, collected from the biodiverse country Vietnam. They were analyzed for their potential do degrade lipids, chitin, cellulose, and xylan (Brandt et al., 2018). Twelve highly active xylan degraders were identified, while two of them were *Aspergillus* strains with high activities in all four investigated assays. By genome sequencing, we compared the phylogenetic relationship and the potential CAZymes of our strain with the CBS 593.65 *A. sydowii* of unknown origin, which was recently sequenced by the Joint Genome Institute (JGI; de Vries et al., 2017). Using zymography coupled with mass spectrometry, we were able to identify three potential xylanases. Two of them were annotated as differing endo-xylanases highly active and heterologously expressed and characterized. These enzymes showed promising activities for sustainable industrial bioprocesses around the enzymatic degradation of xylan-containing biomass.

## Materials and Methods

### Fungus Isolation and Growth Conditions

The *A. sydowii* strain was isolated from shrimp shells in Vietnam according to Brandt et al. (2018). Fungal mycelium was cultured in liquid potato-dextrose-broth (PDB) at 28°C with 150 rpm and used to isolate DNA.

**Figure 1:**
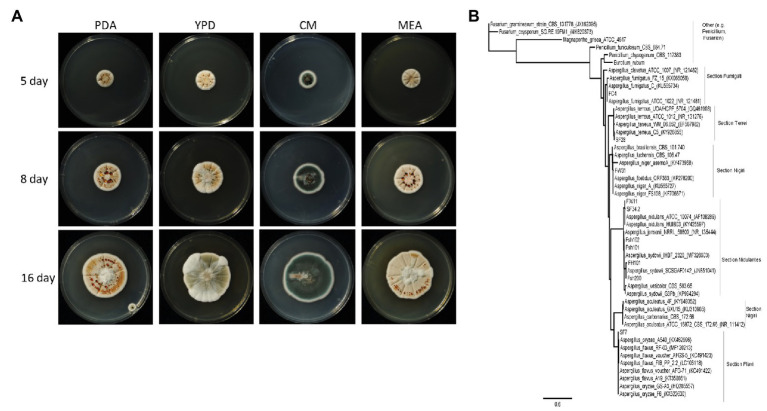
**(A)** Overview of the growth of *Aspergillus sydowii* Fsh102 using four different media. The cultures were incubated at 28°C for 16 days. Pictures were taken after 5, 8, and 16 days. PDA, potato-dextrose-agar; YPD, yeast extract peptone dextrose; CM, complete media; and ME, malt extract agar. **(B)** Phylogenetic tree from *Aspergillus* clades of ITS sequences from the unique fungal strain collection from Vietnam (Brandt et al., 2018) with two strains of *Fusarium* and one for *Magnaporthe grisea*, as well as one for *Eurotium rubum*. Phylogenetic tree highlighting the position of *A. sydowii* isolate and closely related type strains. The tree was inferred from aligned internal transcribed spacers 1 and 2 without adjacent ribosomal genes. Phylogenetic tree was inferred under the maximum likelihood (ML) criterion using the PhyML function in geneious® applying the Jukes Cantor substitution model JC69.

For storage and further experiments, a conidia suspension was used. A 5 mm diameter mycelium piece from potato-dextrose-agar (PDA), was transferred to a new PDA plate and grown in the dark for 5–7 days at 28°C. The conidia were scraped with Drigalski cell spreader and sterile water centrifuged 4°C with 2,693 *g*, washed twice with sterile water, followed by filtering through a 40 μm mesh sieve. Centrifuged at 4°C with 2,693 *g* again for concentration and resuspended in sterile water, aliquoted and stored at −70°C. The fungus was deposited at the DSMZ under the number DSM105790. To investigate mycelial colors, fungal growth was determined on PDA, yeast extract peptone dextrose (YPD; CSH Protocols, 2010), complete media (CM; Leach et al., 1982), and malt extract agar (MEA; Raper and Thom, 1949; Figure 1A).

For the enzymatic evaluation and protein identification, a two-step mycelium cultivation was performed. Mycelium was pre-cultivated in YPD media at 28°C at 150 rpm for 3 days, then washed briefly and dried between filter papers (Whatman, Dassel, Germany). One tenth gram of semi-dried mycelia was incubated with 50 ml of inductive media for 3 days, at 28°C and 150 rpm. The inductive media consisted of a mineral salt media (0.35% NaNO_2_, 0.15% K_2_HPO_4_, 0.05% MgSO_4_ × 7 H_2_O, 0.05% KCl, 0.001% FeSO_4_ × 7 H_2_O) supplemented with 1% (w/v) birch xylan (Sigma Aldrich, Steinheim, Germany).

### Enzymatic Evaluation of the Fungal Supernatants

For the detection of the enzymatic activity of the 24 fungal strains of the collection identified by Brandt et al. (2018), 1.5 ml of the supernatant was incubated with 1.5 ml 2% (w/v) substrate (xylan from birch wood, Sigma-Aldrich, Steinheim, Germany) in 50 mM sodium acetate pH 6.5 in 37°C for 2 h under constant shaking at 300 rpm. Enzyme activity was determined by quantifying the resulting d-xylose originating from xylanase activity by DNS assay. DNS assay was done as described previously (Miller, 1959).

### DNA Isolation and Genomic Sequencing

High molecular gDNA from *A. sydowii* strain were isolated with the CTAB method (Doyle and Doyle, 1990; Richards et al., 1994). The ITS-1/8S rRNA/ITS-2 region was amplified and sequenced with primers ITS1 [F] and ITS4 [F] (Supplementary Table 5) according to White et al. (1990). Sequencing reactions were performed using the ABI Dye Terminator technology according to the manufacturer’s instructions (Applied Biosystems, Foster City, CA, United States). The ITS sequence have been deposited in GenBank with accession number MG098740. An amount 100 μl gDNA solution with a concentration of 2,600 ng/μl were sent to Beijing Genomics Institute (BGI) for sequencing, a 0.8% agarose gel of the isolated gDNA can be found in the Supplementary Material (Supplementary Figure 1). The gDNA for sequencing was prepared as describe by Meyer and Kircher (2010). The library products were sequenced *via* Illumina HiSeqTM 4000.

### Bioinformatic, Phylogenetic, CAZyme, Xylanase Sequence, and Glycosylation Analysis

The genome is available at NCBI with the BioSample number of SAMN08024727. The SOAPaligner (version 2.21) was used to align reads to reference sequence (*A. sydowii* CBS 593.65) and to calculate the average depth and coverage ratio compared to the reference sequence as describe by Gu et al. (2013). Based on aligned results of filtered reads and reference sequence, the single nucleotide polymorphisms (SNPs) and an insertions or deletions of bases (InDels) are annotated.

From the unique fungal strain collection from Vietnam (Brandt et al., 2018), 10 strains from *Aspergillus* as well as ITS sequences from 33 *Aspergillus* spp., two *Penicillium* spp., two *Fusarium* spp., and one *Eurotium rubum* retrieved from MycoBank (Robert et al., 2013), have been used. The phylogenetic tree (Figure 1B) was inferred under the maximum likelihood (ML; Guindon et al., 2010), from sequences of the internal transcribed spacers 1 and 2, aligned with ClustalW (Thompson et al., 1994) with gap open cost of 15 and gap extend cost of 6.66 with free end gaps.

For the CAZyme analysis, the coded regions (CDS) resulting from genomic sequencing were compared with the CAZyme database (Cantarel et al., 2009; Lombard et al., 2014). The annotation of the CAZyme resulted from a combination of the RAPSearch2 search (Ye et al., 2011; Zhao et al., 2012) and HMMER scanning (Finn et al., 2014) described in Hahnke et al. (2015).

**Figure 2:**
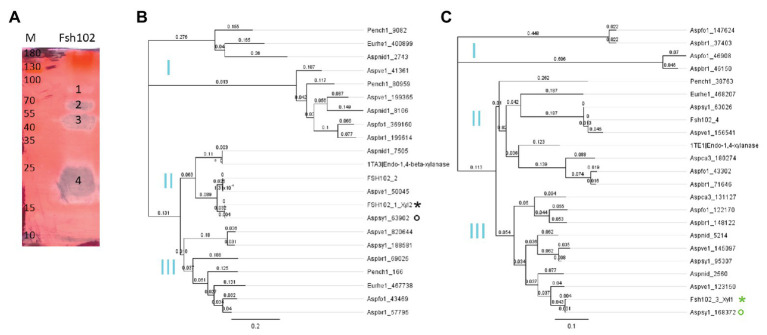
**(A)** Zymogram of xylanase activity using the supernatant of the Fsh102 fungal strain separated by a 12.5% (w/v) SDS-PAGE containing 0.5% (w/v) xylan. The xylan in the gel was stained with 0.1% (w/v) Congo red. Fsh102: the total supernatant of the fungal isolate Fsh102 after 24 h incubation in 1% (w/v) xylan medium. The areas with xylanase activity (1–4) remain unstained. M: Page Ruler Plus Prestained Protein Ladder. The molecular weights of the standard are given in kDa. **(B,C)** Phylogenetic trees of amino acid sequences of selected members of the GH10 **(B)** or GH11 **(C)** families. The global alignment was performed with free end gaps and the cost matrix Blosum62 was used with genetic distance model Jukes-Cantor. The gene are from the JGI MycoCosm. Aspbr1, *Aspergillus brasiliensis*; Aspfo1, *Aspergillus luchuensis* formally known as *Aspergillus foetidus*; Aspnid1, *Aspergillus nidulans*; Aspsy1, *Aspergillus sydowii*; Aspve1, *Aspergillus versicolor*; Aspca3, *Aspergillus carbonarius* formally known as *Aspergillus aculeatus*; Eurhe1, *Eurotium rubum*; and Pench1, *Penicillium chrysogenum*. The gene identified are behind the underline. From the Protein Data Bank (PDB), a candidate for the GH10 or GH11, respectively, was used 1TA3 an 1,4-β-xylanase GH 10 from *Aspergillus nidulans* for the Xylanase II or 1TE1 an Endo-1,4-β-xylanase GH 11 from *Penicillium funiculosum*. For the Fsh102 two candidates are added per GH family (FSH102_1 to FSH102_4), while xylanase II = FSH102_1_Xyl2 (black star) and xylanase I = FSH102_3_Xyl1 (green star). The primers were deduced from the sequences marked with the circles, while the green one was used for the xylanase I or the black for the xylanase II, respectively. Subclades (I–III) are marked in cyan.

For the generation of protein trees the software Geneious version 9.1 was used. The alignment type was global with free end gaps, as cost matrix Blosum62 (Henikoff and Henikoff, 1992) was used with genetic distance model Jukes-Cantor (Jukes and Cantor, 1969). The tree build method was neighbor-joining with no outgroup and a gap open penalty of 12 and a gap extension penalty of 3. Proteins from GH10 or GH11 family, respectively, were downloaded manually from the JGI MycoCosm (Grigoriev et al., 2014), showing high similarity to Fsh102 strain, i.e., *Aspergillus brasiliensis*, *Aspergillus luchuensis*, *Aspergillus nidulans*, *A. sydowii*, *Aspergillus vesicolor*, as more distant strains *E. rubum* and *Penicillium chrysogenum* were used. For Fsh102 strain, the proteins of the GH10 and GH11 families were added. Furthermore, the highest similar structure (mmseqs2 method from the PDB) was downloaded from the Protein Data Bank (PDB) and added, namely 1TA3 an endo-1,4-β-xylanase GH10 from *A. nidulans* (Payan et al., 2004) for xylanase II or 1TE1 an endo-1,4-β-xylanase GH11 from *Penicillium funiculosum* (Payan et al., 2004) for xylanase I, respectively. Separate trees were created for the GH10 or the GH11 (Figures 2B,C). The GenBank files for xylanase I GH11 is BK013306, for the xylanase II GH10 is BK013307, and for a putative exo-1,4-β-xylosidase GH3 is BK013308.

For the O-glycosylation the tool NetOGlyc 4.0 Server (Steentoft et al., 2013) and for the N-glycosylation of the proteins the tool NetNGlyc 1.0 Server (Gupta et al., 2004) were used.

### Zymography and Mass Spectrometry

For zymogram gels, 12.5% SDS-PAGE (Laemmli, 1970) mixed with 0.5% xylan. The supernatants were mixed in equal parts with SDS-PAGE sample buffer and separated in SDS-PAGE. The proteins in the gel were then renatured in refolding buffer [TRIS-HCl 50 mM pH 6.8, 2% (v/v) Triton X-100] for 30 min at room temperature. The gel was washed for a further 30 min in 50 mM TRIS-HCl (pH 6.8) and incubated for one hour in washing buffer (Sodium phosphate 100 mM pH 6.8) at 37°C. Subsequently, unreacted substrate was stained with Congo red and excess dye was removed with 1 M NaCl solution by changing the solution several times. In order to obtain a clear decoloration, the gel was incubated in 70% (v/v) ethanol for a few minutes. For the LC-MS/MS analysis, visible zones of the zymogram gel (Figure 2A) or the SDS gel (Supplementary Figure 5) were cut out and treated following the protocol of Shevchenko et al. (2007) prior to LC-MS/MS analysis. Protein identification was performed *via* analysis by subjecting the tryptic peptides to LC-MS/MS equipped with Acclaim PepMap RSLC C18 column (Thermo Fisher Scientific, Carlsbad, United States). Samples were injected onto a nano-liquid chromatography system (Dionex UltiMate 3000 RSLCnano, Thermo Scientific, Germany) coupled *via* electrospray-ionization (ESI) to a mass spectrometer (MS) equipped with a quadrupole, a linear trap, and an orbitrap (Orbitrap Fusion, Thermo Scientific, Germany). Identification of the proteins from the MS/MS spectra was performed using the search engine Sequest HT and the JGI *A. sydowii* database (de Vries et al., 2017) or the protein sequences of xylanase I or xylanase II from Fsh102. Peptides with a false discovery rate (FDR) of 1% were identified. At least two unique peptides per protein had been found for a reliable identification.

### Cloning of Xylanase Encoding Genes

Mycelium (about 100 mg), grown 3 days in the inductive media, was harvested and grinded using liquid nitrogen. For isolation of total RNA, peqGOLD TriFast™ (Peqlab Biotechnologie GmbH, Erlangen, Germany) was applied. Integrity of the RNA was checked by agarose gel electrophoresis and ethidium bromide staining. cDNA was constructed using the RevertAid H Minus First Strand cDNA Synthesis Kit according to the manufacturer’s instructions (Thermo Fisher Scientific, Carlsbad, United States), the reverse transcriptase was used for first-strand synthesis with random hexamer, as well as for the second-strand amplification of cDNA. PCR primers were deduced from amino acid sequences of xylanase I from the Aspsy1 168372|estExt_Genewise1.C_1_t60491 gene and for xylanase II from the Aspsy1 63902|estExt_fgenesh1_pg.C_1_t20379 gene, found by mass spectrometry detection. Primer design was assisted by the Oligo Calc tool^1^, while the FastCloning method (Li et al., 2011) was used for direct cloning into the expression vector pET28b (BioCat, Heidelberg, Germany). The same method was used for the removal of the secretion signal (Petersen et al., 2011) for both recombinant xylanases enzymes.

### Protein Biosynthesis and Purification

Kanamycin 50 μg/ml was used in the cultivations of *Escherichia coli* as antibiotic. In a typical procedure, *E. coli* BL21 (DE3) cells transformed with pET28b_Xylanase were inoculated in LB medium from an overnight culture (1:100 v/v) and grown at 37°C until an OD_600_ of 0.2–0.5. Protein expression was initiated by the addition of IPTG to a final concentration of 2 mM, while lowering the temperature to 22°C. 17 h after induction, the cells were harvested (4,000 g, 4°C, 10 min) and resuspended in 50 mM sodium phosphate buffer (pH 8). The cells were lysed with ultrasound, using Hielscher UP200H (Hielscher Ultrasonic GmbH, Teltow, Germany) twice for 2 min on ice, 60% amplitude and cycle 0.5, and centrifuged to separate cell debris from soluble protein. The His_6_-tagged xylanases were purified by affinity chromatography using self-packed Nickel-NTA (PureCube Ni – NTA Agarose; Biozym, Hamburg, Germany) columns (Qiagen, Hilden, Germany). In a second step, the xylanases were desalted using Centricon filters of a molecular weight limit of 10 kDa (Merck Millipore, Billerica, USA). The fraction of the affinity chromatography was loaded in the Centricons and washed three times with sodium phosphate buffer (50 mM, pH 8) to remove imidazole. Finally, the purified protein was concentrated to ~2 ml using the same buffer. Purity was evaluated with SDS-PAGE, while protein content was determined using Roti®-Nanoquant protein detection kit (Carl Roth, Karlsruhe, Germany) following manufacturers’ protocol.

### Xylanase Assays

To examine substrate specificity 10 μl of appropriately diluted enzyme solutions was mixed with 40 μl buffer and added to 50 μl substrate solution. The enzymatic hydrolysis was performed in TGradient thermocycler (Biometra, Göttingen, Germany) at different temperatures for 15 min. Followed by the addition of 100 μl DNS solution to generated color formation at 95°C for 10 min. One hundred microliter of these reaction mixtures were measured in the Mithras2 LB943 spectrophotometer (Berthold Technologies, Bad Wildbad, Germany), by the absorbance at 575 nm and correlated to a standard curve for quantification. For each measurement, a negative control without enzyme solution was included. One unit of activity was defined as the amount of enzyme required to release 1 μmol of product equivalent per min in the assay conditions.

To measure the substrate specificity, the xylan was replaced by xylobiose, xylotriose, xylotetraose, xylopentose and xylohexose, arabinan, arabinoxylan, CMC, galactan, and starch, respectively.

Inhibition or enhancement of xylanase activity was determined by applying a range of different compounds to the standard reaction. The following substances were evaluated: NaCl (10 mM, 1,000 mM), MgCl_2_ (5 mM, 100 mM), MnCl_2_ (5 mM), EDTA (2 mM, 50 mM), and DTT (10 mM, 50 mM). Furthermore 5 and 30% methanol, ethanol, acetonitrile, and DMSO, as well as Tween20 with 0.25 and 5%, Triton-X 100 with 0.25 and 5%, and SDS with 1% were evaluated.

To test the temperature range of the enzyme, the standard assay mixture activity was measured at temperatures between 10 and 90°C.

The pH range was determined with 0.1% of xylan from birch wood (Sigma-Aldrich, Steinheim, Germany) applying a broad range of pH from 2.5 to 10. Following buffers were used: glycine HCl (pH 2.5–3.0), citrate (pH 3.0–6.0), phosphate (pH 6.0–8.0), and glycine NaOH (pH 8.5–10.0), all 50 mM. Thermostability and buffer stability were determined by measuring residual activity with the standard assay after preincubation of enzyme samples under the following conditions: temperatures were 4, 30, 50, and 70°C for up to 8 days, in 50 mM sodium acetate buffer pH 4.8 and 50 mM glycine NaOH buffer pH 9 for up to 8 days.

To measure the substrate depended activity xylan concentration was changed in a range from 0.1 to 3.0% using standard conditions. The K_M_, V_max_, and k_cat_ values are calculated using the software GraphPad Prism 8.0.0 (GraphPad Software Inc., San Diego, United States).

The reported activities and standard deviations are the mean of three purifications (from independent cultivations), each measured in triplicates.

### Analysis of Hydrolysis Products by Thin-Layer Chromatography

For the analysis of the hydrolysis products of birch xylan (Sigma Aldrich, Steinheim, Germany), XOS: xylose, xylobiose, xylotriose, xylotetraose, xylopentose, and xylohexose (Megazyme, Wicklow, Ireland) as solutions of 0.1% were used as standards. The 0.1% xylan (50 mM citrate buffer pH 4.8) was analyzed after applying 1 μg xylanase I or xylanase II, respectively, at 30°C. Aliquots from these incubation were taken after 15 min, 60 min, and 17 h, spotted and analyzed on silica-gel G-60 plates F_254_ (20 × 20 cm, Merck). For separation, ethyl acetate/acetic acid/formic acid/distilled water (9:3:1:4; v/v/v/v) was used as mobile phase. After separation, the plates were dried at 80°C for 10 min followed by visualization of the sugars by spraying freshly prepared 0.2% (w/v) orcinol in sulfuric acid/methanol (1:9; v/v) solution and incubation at 80°C for 2 h.

### Creation of Homology Models and Site-Directed Mutagenesis

Homology models for the two xylanases were created using YASARA (ver. 13.9.8). These models were used to determine the active site residues.

The site-directed mutagenesis to prove the active site residues was performed using a modified QuikChange® PCR protocol (Nobili et al., 2013), and the products were transformed in *E. coli* NEB 10 β cells to repair the nicked ends, followed by plasmid isolations to finally transform into BL21 (DE3) for protein expression.

## Results

### Isolation and Characterization of the Fungal Strain Fsh102

By screening a collection of 295 fungi from nine different habitats in Vietnam, prolific universal degrader of lipids, chitin, cellulose, and xylan (Brandt et al., 2018) were identified. We investigated 24 different isolates of these fungi, while the specific enzymatic activity of the cultural supernatant of fungus Fsh102 was 0.61 ± 0.01 U/mg (Supplementary Table 1). For this fungus, isolated from shrimp shells, mycelial growth and pigmentation on different media were monitored (Figure 1A). Depending on the media, pale-white, to orange and up to green colored colonies were identified, which are typical for *Aspergillus* species. Genomic DNA was isolated (Supplementary Figure 1) and the internal transcribed spacer regions one and two were amplified and sequenced (Supplementary Data). Sequencing revealed Fsh102 to be an *A. sydowii* strain (now stored under the number: DSM105790) and the phylogenetic relationship fortifies this results (Figure 1B). The three nearest relatives are all different *A. sydowii* strains, while *A. sydowii* IHB_2320 showing 100% identity with Fsh102. In general, *A. sydowii* is known to have a marine lifestyle, which is in line with the discovery on shrimp shells (Liu et al., 2019).

### Evaluation of the Enzymatic Activity and Identification of Xylanases

By cultivating the fungus in liquid inductive media containing 1% xylan as only carbon source, xylan biopolymer degradation were be observed (Supplementary Figure 2). We, therefore, performed a zymogram, of supernatant from inductive media, to identify the responsible proteins (Figure 2A). The separated zymogram bands were digested and analyzed by protein mass spectrometry (Supplementary Tables 2 and 3). Analyzing the four activity spots from the zymogram by mass spectrometry, 24 proteins were detected in total, from which five of them occurred twice. For the 19 unique proteins, only six putative enzymes were assigned. Five of them are GH family members of which three are predicted enzymes acting on xylan, one exo-xylanase, and two endo-xylanase. We focused on the two putative endo-xylanases, designated as xylanase I (Supplementary Table 4, similar to the JGI automated annotation Aspsy1 168372|estExt_Genewise1.C_1_t60491) and xylanase II (Supplementary Table 4, similar to the JGI automated annotation Aspsy1 63902|estExt_fgenesh1_pg.C_1_t20379). Xylanase II was identified from zymogram band 3, and has a length of 328 amino acids and a predicted mass of 35.43 kDa. Xylanase I was detected from zymogram band 4 with a length of 220 amino acids and a predicted mass of 23.52 kDa. The clearing zone in band 1 was caused by the putative exo-xylanase (Supplementary Table 4, similar to the JGI automated annotation Aspsy1 84747|gm1.1430_g), while the zone 2 presumably originated from a promiscuous endo-glucosidase, because no other enzymes was found inside this zone.

### Bioinformatic Analysis of *Aspergillus sydowii* Fsh102

We used the JGI sequenced strain *A. sydowii* CBS 593.65 (taxid: 1036612) for comparison of with isolate Fsh102. The assembled genome of the *A. sydowii* strain CBS 593.65 has a size of 34.38 Mbp (reference genome). In comparison to this, a sequencing coverage rate of 88.65% was obtained, resulting in a 30.48 Mbp genome of *A. sydowii* strain Fsh102. Compared to the *A. sydowii* CBS 593.65, the Fsh102 has 91,709 total SNPs (0.267%) and 2,829 total InDels (0.008%).

### CAZyme Analysis

The CAZyme analysis of the Fsh102 *A. sydowii* in comparison to selected *Aspergillus* strains and some other ascomycetes can be found in the Supplementary Table 6. In general, it can be stated that the set of extracellular enzymes predicted in Fsh102 was higher compared to the eight *Aspergillus* strains from the database. In the Fsh102 genome, 640 genes coding for different CAZymes were found compared to 612 in the *A. sydowii* reference strain (CBS 593.65). The lowest number was identified for *Aspergillus clavatus* NRRL1 with 384, while the other species rank between 522, for the *Aspergillus aculeatus* ATCC1687, and 597 for *Aspergillus flavus* NRRL3357. The highest number was identified in a *Fusarium oxysporum* strain with 777 putative CAZymes, while *Fusarium graminearum* PH_1 harbors 533 and *Magnaporthe grisea* DS9461 575, respectively. A comparison of Fsh102 and the *A. sydowii* strain sequenced by JGI showed a similar distribution of the CAZymes, reflecting their close phylogenetic relationship. For the Fsh102 strain 310 GHs and 35 GH with additional carbohydrate binding modules (CBMs) were found, while in the JGI strain 297 GHs and 29 GHs with CBMs were present. A difference between both *A. sydowii* strains was found in the GH18 family, which harbors different chitin degrading enzymes, such as chitinases or endo-β-N-acetylglucosaminidases. Enzymes of this family are represented by 20 genes in Fsh102, whereas only 11 GH18 coding genes have been found in the JGI reference strain. Similar differences are found in the GH18 family with additional CBMs, where 13 are predicted in the Fsh102 and only eight in the JGI strain, respectively. In addition, 25 and 29 members of the GH3, family, which consists mostly of exo-acting β-d-glucosidases were identified in Fsh102 and the JGI reference strain, respectively. The Fsh102 has two putative of GH11 candidates, while the JGI strain has three GH11 and both have two GH10 proteins.

### Comparisons of the Xylanases Inside the *Aspergillus* Family

Sequence analysis of xylanase I indicated a membership to GH family 11, while xylanase II belongs to GH family 10. In total, 46 sequences were used for both the protein tress of the GH10 and GH11 families and both families form three subclades (I to III; Figures 2B,C). When considering the protein sequence, both Fsh102 xylanases are very similar to *A. sydowii* CBS 593.65 gene Aspsy1_63902 (xylanase II) or the JGI reference strain gene Aspsy1_168372 (xylanase I). They have a score of 0.004 for both enzymes using the cost matrix Blosum62, indicating that these enzymes are very similar but not identical to the ones in *A. sydowii* CBS 593.65. The differences are 0.45% for xylanase I or 0.3% for xylanase II, when directly compared to the corresponding proteins of the *A. sydowii* CBS 593.65. This difference is higher than the SNPs rate of 0.267%. Xylanase II seems to fall in the same subclade (II), as the nearest crystallized protein, 1TA3 from *A. nidulans*. Xylanase I is found in a different subclade (III) then their nearest crystallized protein 1TE1 from *P. funiculosum* which belongs to subclade II, both according to the PDB. Furthermore, the GH10 candidates of the Fsh102 are more similar than the GH11 candidates, as both proteins of the GH10 belongs to the same subclade II, while the GH11 do not (subclade II and III).

### Glycosylation Prediction

The prediction of the glycosylation of xylanase I indicates that the enzyme is N-glycosylated at one site and O-glycosylated at two positions. For xylanase II, two N-glycosylations and two O-glycosylations, both O-glycosylations within the sequence of the signal peptide, were predicted (Supplementary Figure 11).

### Cloning of the Xylanase Genes and Purification of the Two Endo-Xylanases

The ORF of xylanase I has a length of 729 bp and is organized in two exons found on contig 108. The ORF of xylanase II is considerably longer, consisting of 1,541 bp, which are organized in 11 exons, found on contig 55 (Supplementary Table 7). Xylanase I has a putative 19 amino acid long signal peptide, which was predicted from sequence analysis using the SignalP 4.1 Server (Petersen et al., 2011; Supplementary Figure 3). Not so obvious was a putative 21 amino acid long signal peptide for the xylanase II, because the S-score, indicating the end of the putative signal peptide after position 20, whereas the Y-score, which indicate the cleave position, was below the given threshold (Nielsen, 2017; Supplementary Figure 4). The expression of both enzymes including their putative signal peptides resulted in the production of inactive proteins. Therefore, FastCloning (Li et al., 2011) was used to remove the putative signal peptides from both enzymes and expression was performed using an inducible system. The specific activity of the crude extract of xylanase I was 33.7 U/mg, while the elution fraction 1 had a specific activity of 181.1 U/mg, resulting in 55% yield and a purification factor of 5.4 (Supplementary Table 8). For xylanase II, the activity of the crude extract was 57.1 U/mg, already almost twice as high as xylanase I crude extract. The specific activity of the elution fraction 1 of xylanase II was 106.1 U/mg resulting in a yield of 27% and a purification factor of about two, but the elution fraction 2 corresponded to a specific activity of 121.5 U/mg. Further analysis was performed using the elution fraction 1 of both enzymes. In Supplementary Figure 5, the electrophoretic separation of different protein fractions is displayed, showing that a large portion of the total protein is found in the insoluble part for both proteins. Additionally, it was observed that the binding of xylanase I to the Ni-NTA agarose was tighter, if compared to xylanase II, as about 60% of the xylanase II are lost during cleaning procedure (flow through and wash fraction), while only about 36% of xylanase I was lost under the same conditions. The heterologous expressed xylanases were verified by liquid chromatography with coupled mass spectrometry (LC/MS-MS) of the corresponding protein bands from the SDS gels (Supplementary Figure 5). For xylanase I 47.68% (three different peptides) and for xylanase II 48.83% (five different peptides) of their total sequences were found (Supplementary Figure 9). These prove the expression of the targeted enzymes.

### Substrate Specificity of Xylanase I and Xylanase II

Both xylanases showed high specificity against xylan compared to other substrates (Supplementary Figure 6A). Other hemicellulose-related substrates such as arabinoxylan or arabinan were hardly converted (less than 8% relative activity) and similar weak activities were observed using starch, galactan, or carboxymethylcellulose as substrate. Moreover, the enzymes showed little activity against xylopentose, 21.5 ± 11.0% for xylanase I and 13.5 ± 1.3% for xylanase II, and xylohexose with relative activities ranging from 16 ± 5.4 to 15 ± 1.0% for xylanase I and xylanase II, respectively. Interestingly, xylanase II showed low (12.4 ± 1.4%) but significant activity against xylotriose, but no activity in case of xylobiose or xylotetraose. Xylanase I was not able to hydrolyze XOS shorter than xylopentose or their conversion was below detection limit.

For both enzymes, we found that higher substrate concentrations resulted in higher specific activities (Supplementary Figure 6B), with an even more pronounced effect for xylanase II. The enzyme xylanase I showed about 12-fold higher activity at 3% xylan compared to 0.1% xylan, whereas xylanase II increased its activity roughly 8-fold. The K_M_ values are 20.68 ± 8.53 and 6.71 ± 3.14 g/L for xylanase I or xylanase II, respectively, while V_max_ are 3.12 ± 0.74 and 0.94 ± 0.16 mM/min. Turnover number, k_cat_, was found to be 439.98 s^−1^ for xylanase I and 199.44 s^−1^ for xylanase II, resulting in an catalytic efficiency, k_cat_/K_M_, of 21.28 L*g^−1^*sec^−1^ and 29.72 L*g^−1^*sec^−1^.

### Influence of Temperature and pH on Xylanase Activities

Xylanase I was completely functional in the range of 30–50°C (100 ± 11 to 109 ± 12%) and retained roughly half of its activity at 70°C (47 ± 3%; Supplementary Figure 7A). Xylanase II almost doubled its activity at 50°C compared to 30°C, and retained 27 ± 1% activity at 70°C (Supplementary Figure 7A). The best assay temperature would be 50°C, nevertheless for better comparability 30°C was used. The optimal pH for xylanase I activity was 4.8, and this enzyme showed more than 70% relative activity in a mildly acidic to neutral pH range (pH 4.5–7; Supplementary Figure 7B). Xylanase II activity at pH 4.8 was set to 100% because all measurements were performed at pH 4.8, but highest activity were detected at pH 7.5 with 123 ± 12%. This enzyme has also a much broader range, from 4.5 to 8.5, at which activity remained higher than 85% (Supplementary Figure 7B).

### Temperature, pH, and Solvent Stability of the Xylanases

**Figure 3:**
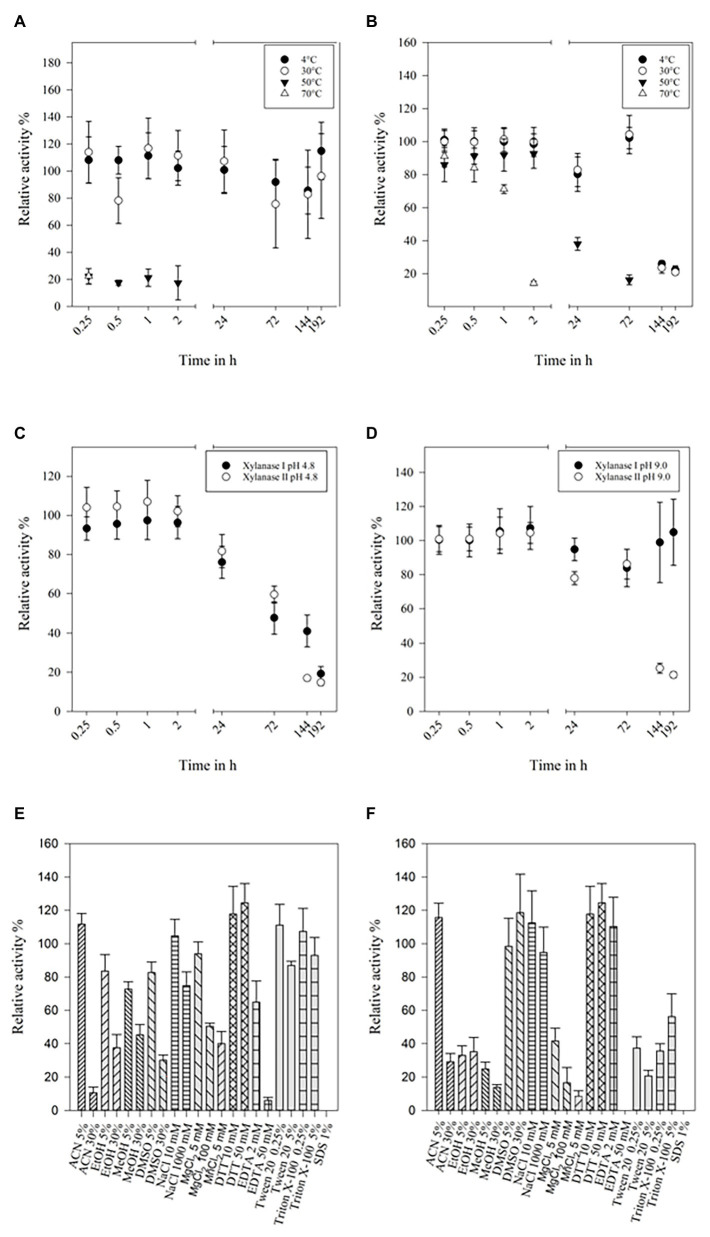
Temperature stability of xylanase I **(A)** and xylanase II **(B)**, buffer stability of xylanase I and xylanase II at pH-values 4.8 **(C)** and 9.0 **(D). (A)** Relative activity of xylanase I at 4, 30, 50, and 70°C over a time of 192 h with 30°C set to 100%, **(B)** relative activity of xylanase II at 4, 30, 50, and 70°C over a time of 192 h with 30°C set to 100%, **(C)** relative activities of xylanase I and xylanase II stored in citrate buffer pH 4.8 at 4°C over a time of 192 h with 0.25 h are set to 100%, and **(D)** relative activities of xylanase I and xylanase II stored in glycine NaOH buffer pH 9.0 at 4°C over a time of 192 h with 0.25 h set to 100%. Relative activity of the xylanase I **(E)** and xylanase II **(F)** applying solvents and salts. The following solvents and salts were used: 5 and 30% ACN, 5 and 30% EtOH, 5 and 30% MeOH, 5 and 30% DMSO, 10 and 1,000 mM NaCl, 5 and 100 mM MgCl_2_, 10 and 50 mM DTT, 2 and 50 mM EDTA, 0.25 and 5% Tween 20, 0.25 and 5% Triton X-100, and 1% SDS. One hundred percent are set applying no salts or solvents.

Storing at 4°C did not affect the activity of xylanase I, even after 8 days. If the enzyme was stored at 30°C only a small impact on activity was observed after the third day (>80%; Figure 3A). The enzyme showed 10–20% residual activity after heating it at 50 or 70°C for 15 min. Xylanase II maintained clearly more than 85% activity after incubation at 4, 30, or 50°C for 2 h. Heating the enzyme at 70°C for 2 h resulted in 15% residual activity. After prolonged incubation of 24 h at 50°C, the remaining activity was 40%, while storing at 4 or 30°C more than 85% of the activity was preserved for up to 72 h (Figure 3B). Additionally, the stability in two buffers (pH 4.8 and pH 9) at 4°C was investigated. At pH 4.8, the activity of both xylanases was stable for 2 h and then gradually decreasing to about 20% after 192 h (8 days; Figure 3C). Both enzymes are more stable, if they are stored at pH 9, displaying 80–100% activity for up to 72 h (Figure 3D). The activity of xylanase II was decreasing after that time point, while xylanase I activity was stable (at about 100%) until the end of the measurement (192 h).

The impact of salt ions and modifying reagents on enzyme activity were determined, too (Figures 3E,F). Concentrations up to 5% (v/v) of the different organic solvents used in this study were not affecting xylanase I activity (80–110% residual activity), while 30% (v/v) of the solvents decrease its activity to 10, 40, 40, and 30% for ACN, EtOH, MeOH, and DMSO, respectively. Low concentrations of NaCl (10 mM) and MgCl_2_ (5 mM) did not impair activity, while high concentrations (1 or 0.1 M, respectively) resulted in an activity loss of up to 30%. Almost no effect was observed when using up to 5% (v/v) Tween 20 or 5% (v/v) Triton-X 100. High concentrations of EDTA (50 mM) inhibited the activity as SDS did (Figure 3E). The enzyme activity of xylanase II displayed also a high stability in different solvents, such as 5% ACN, 5% or 30% DMSO, or different concentration of additives like NaCl (10 or 1,000 mM), DTT (10 or 50 mM), and ETDA (2 mM), did not affect the enzymes activity (Figure 3F).

### Analysis of Hydrolyzed Products

**Figure 4:**
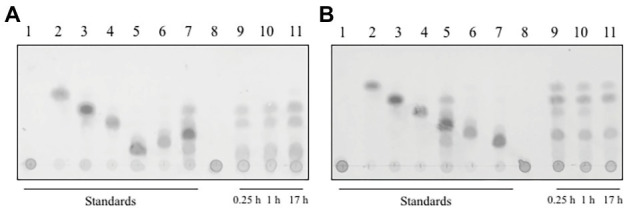
Thin layer chromatography of the reaction products of **(A)** xylanase I and **(B)** xylanase II on a silica gel plate, with a running time of 40 min in organic solvent. The plate was incubated with 0.2% (w/v) oricin at 80°C for 40 min. The produced sugars are visualized as dark spots. **(A)** Lane 1: xylan, lane 2: xylose, lane 3: xylobiose, lane 4: xylotriose, lane 5: xylohexose, lane 6: xylopentose, lane 7: xylotetraose, lane 8: xylanase I + xylan after 0 h incubation, lane 9: xylanase I + xylan after 0.25 h incubation, lane 10: xylanase I + xylan after 1 h incubation, and lane 11; xylanase I + xylan after 17 h incubation. **(B)** Lane 1: xylan, lane 2: xylose, lane 3: xylobiose, lane 4: xylotriose, lane 5: xylotetraose, lane 6: xylopentose, lane 7: xylohexose, lane 8: xylanase I + xylan after 0 h incubation, lane 9: xylanase I + xylan after 0.25 h incubation, lane 10: xylanase I + after 1 h incubation, and lane 11: xylanase I + xylan after 17 h incubation.

In order to analyze the reaction products of the depolymerization of xylan by both xylanases, we used TLC on a silica gel plate and stopped the reaction at different time points (Figure 4). The main products of the hydrolysis of xylan by xylanase I (Figure 4A) were xylobiose, xylotriose, and xylohexose, which are visible after 15 min of incubation. Additional spots between the starting point and the spot for the xylohexose might derive from higher polymerized XOS. During the course of the conversion, the product profile changed slightly, visible by a small spot corresponding to xylose after 17 h. Interestingly, xylanase II generated a different reaction profile (Figure 4B). Directly after the start of the reaction the monosaccharide xylose can be detected, as well as xylobiose, xylotriose, xylopentose, and some higher polymerized XOS. Interestingly, the reaction of xylanase II does not yield xylohexose, and the spot corresponding to xylotriose is disappearing over the course of the reaction. Xylotetraose is neither in the enzymatic reaction of xylanase I nor xylanase II observable, indicating that either no xylotetraose is formed during the reaction or a very rapid turnover of this tetrameric sugar takes place. Due to the determination of the substrate spectra, it is unlikely that a very rapid turnover of this sugar tetramer is performed. These findings underlining the need for cooperation between the two non-redundant xylanases for an efficient degradation.

### Active Site Variants of Xylanase I and Xylanase II

**Figure 5:**
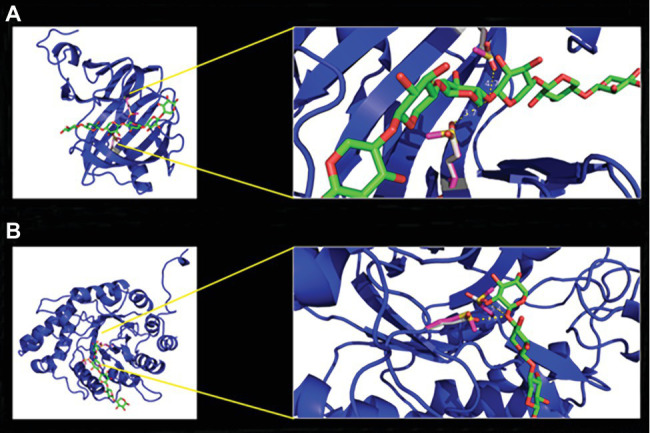
Homology models of xylanase I **(A)** and xylanase II **(B)** with enlargement of the active center. The models were calculated with the program YASARA using the protein database PDB. The models were displayed with the program PyMol. The enzyme backbones are colored in blue, the xylooligosaccharides (XOS) carbon atoms in green, oxygen in red, active site residues glutamic acid carbon atoms in gray, methionine carbon atoms of the variants in pink, and in yellow is the sulfur.

An acid-based catalyzed mechanism is assumed for both enzymes. One residue is acting as a nucleophile, while the other serves as a general acid/base residue. This activity follows a Koshland double-displacement mechanism (Withers et al., 1986; Gebler et al., 1992). To verify this theory, we mutated the identified residues and generated two single mutants and one double mutant for each enzyme. As a replacement for the two glutamate residues, methionine was chosen, based on its similar steric and spatial needs. In the case of xylanase I, the glutamic acid on positions 97 and 188 and for xylanase II the glutamic acids on the positions 155 and 241 were replaced by methionine. For both enzymes, two single and one double mutant were created. The enzyme xylanase I is predicted to exhibit a typical β-jelly roll fold (Figure 5A), while xylanase II has a TIM barrel-(β/α)_8_ fold (Figure 5B), which is in line with the GH family and GH-A clan assignment. The active sites of the enzymes are depicted in the magnified section of Figure 5 (right part). For the xylanase I the distance between the active site glutamic acids is 3.7 and 4.7 Å and for xylanase II we observed 3.0 and 5.4 Å. The activities of the mutated xylanase I and xylanase II were determined to verify that the active site residues were matched correctly. The xylanase I variant E87M displayed 4 ± 0.5%, the variant E188M 13 ± 4.0% and the double mutant E97M/E188M 4.0 ± 0.5% relative activity (Supplementary Figure 8A). The xylanase II variant E155M displays 9.0 ± 2.4%, the variant E241M 7.0 ± 0.6% and the double mutant E155M/E241M 10.0 ± 0.9% relative activity (Supplementary Figure 8B). The purification of different heterologous expressed variants can be found in Supplementary Figure 10.

## Discussion

In the present study, we report the assignment of Fsh102, DSMZ 105790, to the genus *Aspergillus* of the section Nidulantes and classify it as *A. sydowii*, by comparing our genomic data with available genomic information of the *A. sydowii* CBS 593.65, as well as an ITS based sequences analysis with other *Aspergillus* spp.

In comparison to our data, similar coverage and identity were found by a report of two strains of *A. carbonarius*, which had a sequence similarity higher than 97.2% and a coverage rate of more than the 84% compared to the non-repetitive reference genome (Cabañes et al., 2015). The genome size of Fsh102, with approximately 34 Mbp, is in the range of *Aspergillus* species (29–37 Mbp) and most of the genomes of Eurotiales reported to date are in that range (Yang et al., 2016). Especially when comparing the *A. sydowii* genome the section Nidulantes strains, like *A. nidulans* with 30.48 Mbp or *Aspergillus versicolor* with 33.13 Mbp, a slightly larger genome size become obvious. One possible explanation for the large genome size may be the variability of *A. sydowii* fungus to grow pathogenic on animals like corals or humans, as well as to live saprophytic on dead plant matter (Alker et al., 2001; Hallegraeff et al., 2014; Liu et al., 2019). A close relative, *A. carbonarius* ITEM 5010 has a genome size of 36.3 Mbp, making it slightly larger than the *A. sydowii* genome CBS 593.65 genome. Next to the size, the frequency of SNPs and InDels in a genome is an interesting marker. By comparing different *A. carbonarius* strains with our reported analysis of Fsh102, which is based on the *A. sydowii* reference genome, the total number of SNPs was found to be lower (52,661), while the number of InDels was increased (7,567) if the *A. carbonarius* strains were compared to the *A. sydowii* strain analysis.

The wild type extracellular xylan degrading activity of the fungal strain Fsh102 is comparable to published *A. sydowii* SBS 45 (Nair et al., 2008). Other fungi are able to generated similar or higher xylanase activity, like a *F. oxysporum* isolate with 0.41 U/mg (Christakopoulos et al., 1996) or *Trichoderma viride* Fd18, which was reported to be 1.80 U/mg (Ja’afaru, 2013), but the wild type activity of our strain is very comparable to the other reported fungi.

By analyzing the CAZyme prediction in more detail, it was found that Fsh102 is more similar to *A. sydowii* CBS 593.65, while that the total number of CAZyme genes is higher, if compared to other species of this genus. This underlines the general hypothesis that *A. sydowii* needs a broad portfolio of extra-cellular enzymes for its variable lifestyle. These differences are most likely an adaptation to the habitat, while the origin of the JGI *A. sydowii* is not known, Fsh102 was isolated from shrimp shells, which is mirrored in the distribution of the CAZymes. As can be seen in the higher number of chitin degradation enzymes of the Fsh102, which was isolated from aquatic source, compared to the *A. sydowii* reference strain, which has more enzymes responsible for the degradation of (hemi-)cellulose, so it could be speculated that the CBS strain originated from a terrestrial source. Until now, xylanase activities were found in six GH families (5, 7, 8, 10, 11, and 43), but the main focus of biotechnological research is centered on two of the xylanase containing GH families 10 and 11 (Collins et al., 2005). The exo-xylanase (GH3) was not investigated in this work, as we focused on the first steps of xylan degradation. The analyzed xylanase I belongs to the GH11 family, while xylanase II is an enzyme of the GH10 family. The JGI strain has two GH10 and three GH11 enzymes, Fsh102 encodes two members of both. It is striking that only one of these two GHs was found in the zymogram using Fsh102 supernatant, as activities in both families are predominantly related to xylan depolymerization. The lack of observed enzymes of GH10 or GH11 family members can have different reasons. The four most likely are

- The different members are induced differently dependent on growth conditions like various kinds of xylan containing substrates or temperatures. This behavior was observed for *Aspergillus niger* E-1, which encodes different xylanase (GH10 and GH11) in the genome, while only three of them were identified by MS if this fungus was grown on beech wood xylan (Takahashi et al., 2013).
- The members of the GH10 and GH11 family may have different enzymatic activities, but this is unlikely because both classes are extensively studied. There are, at least, 127 GH10 enzymes and 173 GH11 enzymes with a demonstrated activity on xylan described in the literature and only in the case of GH10 different activities were reported. In the GH10 family also endo-β-1,4-xylanases (EC 3.2.1.8), endo-1,3-β-xylanases (EC 3.2.1.32), and cellobiohydrolases (EC 3.2.1.91) are known, while GH11 members are considered to be monospecific (Davies and Henrissat, 1995; Paës et al., 2012; Chakdar et al., 2016).
- The peptides generated by the tryptic digestion were not ionisable. Although this is unlikely, as ESI method is capable of ionizing the majority of peptides and the MS/MS device has a very high mass resolution thereby lowering the detection limit further, it cannot be ruled out.
- The variation within the GH protein families is high in general. Different members of the GH10 subclass belong to different subclade of the protein trees (Figure 2C) and the GH11 member (FSH102_2) showed more similarity toward an enzyme from *A. versicolor* (Figure 2B) than the JGI counterpart. It can be speculated that the predicted GH10 and GH11 enzymes have with different substrate specificities, because this part of the protein tree is more widely dispersed.

To the best of your knowledge, this report describes for the first time that enzymes of the *A. sydowii* were heterologous expressed in high yield, by using a prokaryotic *E. coli* system. An activity of xylanase I and xylanase II with 181.1 and 121.5 U/mg was obtained in their N-terminal truncated versions, while only insoluble proteins were formed, if expressed with the corresponding fungal N-terminal secretion signal. Frequently, more complex eukaryotic expression systems, like *Komagataella phaffii* (formerly known as *Pichia pastoris*) or *Yarrowia lipolytica*, have to be used to express eukaryotic xylanases from *A. niger*, *Trichioderma harzianum*, *Hypocrea orientalis*, or *Trichioderma reesei* (He et al., 2009; Wang et al., 2014; Li et al., 2018) as they have the advantage of correct N‐ and O-glycosylation. The glycosylation can have different effects like enhancement of solubility (Solá and Griebenow, 2009), increased activity, as shown for the endo-cellulase IIa from *Penicillium verrucosum* (Dotsenko et al., 2016), or enhanced stability (Sarkar and Wintrode, 2011). On the other hand, there are a number of reports showing the expression of xylanases in *E. coli* (Basaran et al., 2001; Le et al., 2011) but having the disadvantage of either being periplasm bound to the *E. coli* or having low activity (about 30 U/mg). The two presented xylanases exhibit comparable activity to other heterologous expressed xylanases. Two reports from *Komagataella phaffii* expression studies yielded 175 U/mg from a xylanase originating from an *A. niger* strain (Liu et al., 2006) or 123 U/mg from a strain of *Bacillus licheniformis* (Liu and Liu, 2008), but there are also studies reporting exceptional high enzymatic activity like a *T. reesei* xylanase with 746 U/mg (He et al., 2009). It, therefore, cannot be ruled out that the activity, of the xylanases reported in this study, could be much higher if a eukaryotic expression system would have been used. The xylanase from previously mentioned *T. reesei* had about 12-fold reduced activity if expression was performed in *E. coli* instead of *P. pastoris* (He et al., 2009).

In the publication of Nair et al. (2008), two xylanases of a crude culture filtrate of *A. sydowii* SBS 45 were partially characterized, but no heterologous expression was performed. Comparing the molecular weights of the enzymes with our study, a good match can be found for both of the heterologous expressed xylanases of the *A. sydowii* Fsh102. Xylanase I, reported in this study, shows a clearing zone in the zymogram in the region of 15 and 25 kDa (Figure 2A Band 4), which is similar to the xylanase I of SBS 45 which has a molecular weight of 20.1 kDa. For xylanase II of Fsh102, a weight of 37.1 kDa was calculated (Supplementary Figure 5B, 39.2 kDa, including signal peptide), but the zymogram showed a clearing zone in the region of 45 kDa (Figure 2A Band 3). This shift can be explained by the glycosylation of the enzyme (2 N-glycosylations shown in Supplementary Figure 11). The xylanase II from *A. sydowii* SBS 45 was reported with 43 kDa based on an SDS-PAGE analysis, displaying a similar observed size as xylanase II of Fsh102. The purification quotient of 5.41 for xylanase I and 1.96 for xylanase II is comparable to the purification quotient of 5.3 and 1.9 for two xylanases of *Trichoderma inhamatum* (Silva et al., 2015). To increase the protein yield during the purification process, it is possible to use an extended His_6_-tag, a sequence extended by six histidine molecules. This led to a better binding of the neurotensin membrane receptor to the column and thus to a better purification performance of the protein (Grisshammer and Tucker, 1997).

The substrate specificity study of the purified enzymes reveals that both enzymes have next to their xylanolytic activity, a weak promiscuity. An activity of around 8%, compared to the xylanase activity, was observed for the action on other (bio)polymers like cellulose, identified by carboxymethylcellulose as substrate or arabinan, which consists of about 70% arabinose α-(1–5) linked with 20% galactose and other sugars (Supplementary Figure 6). Generally, xylanases are very specific as shown for the *T. reesei* strain Rut C-30 endo-xylanase Xyn2 of the GH family 11 (He et al., 2009), which has about 1% activity for CMC and the same was shown for a GH10 xylanase from *Caldicellulosiruptor bescii* (An et al., 2015). This weak but existing promiscuity of our xylanases is advantageous, because the use of our xylanases could reduce the need to add other enzymes in a rye-based diet, in food preparation or during the treatment of agricultural wastewater, which both contains different biopolymers. Often, higher xylanase activities are observed if higher xylan concentration were applied during the fermentation by wild type organism like for *Anoxybacillus kamchatkensis* (Yadav et al., 2018). For a better comparison, 0.1% substrate concentration was used for almost all experiments, but higher concentrations yielded higher activities. Around 12‐ and 7-fold higher activities were observed for xylanase I and xylanase II, if 3% substrate was used. Based on the data available, it can be assumed that for xylanase I, a substrate concentration of 2.5% provides the highest activities, while for xylanase II no possible substrate inhibition was observed in the measured range. Similar behavior could be observed for the heterologous expressed XynDZ5 GH10 xylanase from *Thermoanaerobacterium* sp., where three different xylan sources were used, showing about 10-fold increase of activity if 30 g/L (3%) was used instead 0.3% (Zarafeta et al., 2020). Substrate affinity of xylanase II 6.71 ± 3.14 g/L was comparable to literature values of 0.7–6.6 g/L (Li et al., 2006, 2010; Sharma et al., 2010; Ding et al., 2018; Yadav et al., 2018), while xylanase I showed a reduced affinity with a K_M_ of 20.68 ± 8.53 g/L. Although the substrate affinity of xylanase I is reduced, the maximal velocity 3.12 ± 0.74 in mM/min was quite good compared to literature values of 0.067, 1.29, 1.64, and 9.17mM/min. Xylanase II exhibits a maximal velocity of only 0.94 ± 0.16 mM/min, which is also reflected in a relative low turnover number of around 200 per second, while literature values reach up to 7,000 per second. These numbers depend entirely on the substrate used and others substrates might have increased the values what would be especially relevant for the catalytic efficiency, which was found to be between 20 and 30 for xylanase I and II. Here, literature values of around 150–1,000 show an optimization potential for the identified xylanases (Li et al., 2006, 2010; Sharma et al., 2010; Ding et al., 2018).

When comparing the cleavage products of both xylanases, it is noticeable that both endo-xylanases are able to cleave short XOS. Both enzymes have 10–20% activity comparing to birch wood xylan, when substrates with a chain length from five xylose units to six units are used. The enzyme xylanase II has a rather uncommon exception; it is able to degrade xylotriose with 9% activity but not xylotetraose. While other researchers found that, the shortest XOS converted by GH10 or GH 11 xylanases are the xylotriose or xylotetraose longer XOS (Supplementary Table 10; Kolenová et al., 2006; Takahashi et al., 2013; Li et al., 2018; Han et al., 2019).

Both xylanases have mesophilic temperature preferences and show an optimum temperature at 50°C (Supplementary Figure 7A). In addition to their broad temperature profile, both enzymes have a broad pH range, xylanase I 4.5–7.0 and xylanase II 4.5–8.0 (Supplementary Figure 7B). This is similar to different endo-xylanases from other *Aspergillus* strains which have their maximal activities between 42 and 60°C, and a pH range of 4.0–7.0 (Teixeira et al., 2010), or more specifically with *A. sydowii* SBS 45 (Nair et al., 2008) or a GH10 xylanase of *A. niger* E-1 (Takahashi et al., 2013) both having an optimum between 50 and 55°C. The enzymes from Fsh102 show moderate to good activities at 10°C, granting the possibility to degrade xylan at low temperatures for food applications in fruit juice clarification, as it is nowadays mostly performed at mesophilic temperatures between 27 and 43°C (Sharma et al., 2017).

When comparing the temperature stability of both xylanases, it is noticeable that both xylanases have a high stability for up to at least 3 days at lower temperatures. Especially, xylanase II has also a good stability at high temperatures making it competitive toward some of the best xylanases identified so far (Supplementary Table 9). Similar is true when comparing pH stabilities although long time data are scarcely reported (Supplementary Table 9).

The temperature stability results are in line with the investigations of the effect of additional salts and solvents to the enzymatic reaction. In general, xylanase I is more resilient than xylanase II. By comparing the salt tolerance to GH10 xylanases from *A. niger* strain C3486, similarities to the studied xylanases were found. In both cases, low concentrations of Mg^2+^ ions cause no inhibition, but low Mn^2+^ concentrations do (Yang et al., 2010). Interestingly, Mn^2+^ ions are not inhibiting the two *A. sydowii* SBS 45 xylanases, but EDTA is reducing the activity to only 9 and 30%, while both Fsh102 xylanases are more tolerant to EDTA retaining 70 to 110% activity, respectively. The SBS 45 xylanase 2 is moderately inhibited by SDS to 30%, while the Fsh102 xylanase II did not show any residual activity after SDS addition. Reducing agents, like DTT (for Fsh102) or β-Mercaptoethanol (for SBS 45 xylanases), do not affect the enzymes as no disulfide bonds are present. By comparing the enzymes to a xylanase from *A. niger* DSM 1957 (Do et al., 2009), which is stated to be resistant to methanol, ethanol, isopropanol, and acetone up to 30 (v/v)%, or to *Aspergillus awamori* VTCC-F312 xylanase with has a residual activity of 63–86% by 30 (v/v)% solvents (Do et al., 2012), the Fsh102 xylanases can be classified as moderate solvent stable, which is necessary for the use some pulp and paper processing like the organosolve process (Brosse et al., 2019).

The enzymatic reaction for both xylanases is performed using a nucleophile and acid-base catalyst, as two glutamate residues are essential as shown by mutation analysis performed in the presented study. All single and double mutants had a residual activity between 5 and 10%, which is relatively high, if compared to other studies, which are mostly performed with bacterial xylanases like *Streptomyces lividans* or *Bacillus circulans*, whereas the reduction is higher than 10^3^ with up to no xylanolytic activity (Ko et al., 1992; Moreau et al., 1994; Wakarchuk et al., 1994). It might be the case that acidic residues structurally close to the catalytic active residues can replace those originally catalytic active residues to perform hydrolysis, although with lower efficiency. If this taken into account, in a reverse study, were amino acids in 12 Å distance from either of the two catalytic amino acids were replaced, resulting in about 12% residual activity for the worst variant, gives one possible hint that structural close residues to the active site residues have an influence on the catalysis (Bai et al., 2015). A similar argument is given by the fact that in a xylanase originating from *Bispora* sp. MEY-1, two non-polar amino acids near the active site residues were mutated to acidic residues resulted in an increase of the specific activity up to 1.3 fold (Wang et al., 2017).

## Conclusion

In this study, we trace the whole biotechnology pipeline, from strain isolation, activity-based identification of the enzymes by mass spectrometry, heterologous expression to characterization of two xylanase from *Aspergillus sydowii*. Due to their differences in activity, hydrolysis products and stability, these enzymes are non-redundant xylanases and their characteristics provide an interesting starting point for directed evolution. Those robust enzymes, are tolerant toward mesophilic temperatures, acting in a broad pH range and are stable toward organic solvents, detergents and salts, which granting the possibility for food applications in fruit juice clarification or in the bakery or the pulp and paper processing industry.

## Data Availability Statement

The datasets presented in this study can be found in online repositories. The names of the repository/repositories and accession number(s) can be found in the article/Supplementary Material.

## Author Contributions

SB performed DNA isolation, ITS-PCR, BLAST ITS sequences, plate-based screening, and pre cultivation of the fungi. BE measured the activities of the supernantants. TN collected the fungal sample. RH analyzed the ITS-data and generated the phylogentic tree and made the CAZyme analysis. MR annotated the Fsh102 genes. SH and HS performed and analysis the mass spectrometry. WS started the project. MG organized the lab work, coordinated the progress between the different institutions, and evaluated the data. All authors contributed to the article and approved the submitted version.

### Conflict of Interest

The authors declare that the research was conducted in the absence of any commercial or financial relationships that could be construed as a potential conflict of interest.

## Footnotes

^1^http://biotools.nubic.northwestern.edu/OligoCalc.html
