# Supplementary material for: Aspergillus sydowii: Genome Analysis and Characterization of Two Heterologous Expressed, Non-redundant Xylanases
Source: Front Microbiol. 2020 Sep 18;11:2154. doi: 10.3389/fmicb.2020.573482 (PMC7531221; doi:10.3389/fmicb.2020.573482)
Supplement: Supplementary file 1 [file Data_Sheet_1.docx]

Supplementary Material

M

Fsh102

Fsh102

# Supplementary Data

The amplified and sequenced ITS-region with primers ITS1 [F] and ITS4 [F] (Supplementary Table 5) according to White (White *et al*., 1990). Sequencing reactions were performed using the ABI Dye Terminator technology according to the manufacturer’s instructions (Applied Biosystems, Foster City, CA, USA). The ITS sequence have been deposited in GenBank with accession number MG098740.

AAATGGTTGGAGACGTCGGCTGGCGCCCGGCCGGCCCTAGTCGAGCGGGTGACAAAGCCCCATACGCTCGAGGACCGGACACGGTGCCGCCGCTGCCTTTCGGGCCCGTCCCCCGGGGGGGACGACGACCCAACACACAAGCCGGGCTTGATGGGCAGCAATGACGCTCGGACAGGCATGCCCCCCGGAATGCCAGGGGGCGCAATGTGCGTTCAAAGACTCGATGATTCACTGAATTCTGCAATTCACATTACTTATCGCAGTTCGCTGCGTTCTTCATCGATGCCGGAACCAAGAGATCCATTGTTGAAAGTTTTGACTGATTTTATATTCAGACTCAGACTGCATCACTCTCAGGCATGAAGTTCAGTAGTCCCCGGCGGCTCGCCCCCGAGGGGGTTCCCCGCCGAAGCAACAGTGTTAGGTATTCACGGGTGGGAGGTTGGGCGCCCGGAGGCAGCCCGCACTCAGTAAT

# Supplementary Figures and Tables

## Supplementary Figures

**
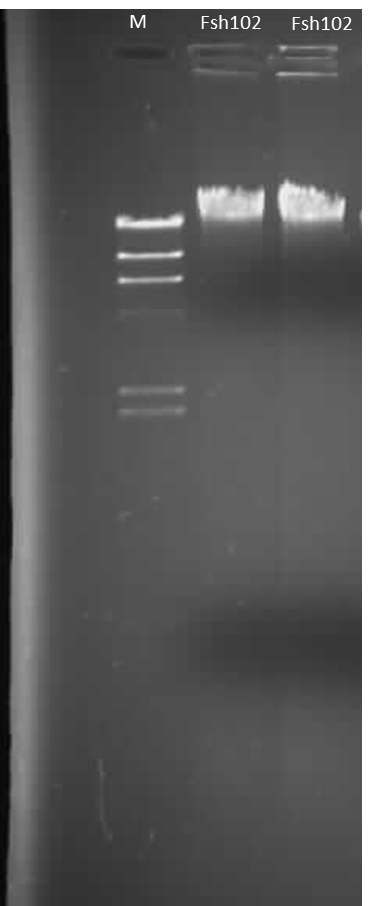
**

**Supplementary Figure 1:** Agarose gel 0.8% (w/v) of the isolated gDNA from *Aspergillus sydowii* Fsh102 strain, according to the CTAB method. gDNA (5 μL) were mixed with the 6 x loading buffer (0.25% (w/v) % xylene cyanol, 0.25% (w/v) bromophenol blue, 30% (v/v) Glycerol, TBE [Tris-Borate-EDTA] (ratio 1:6), loaded into the gel pockets and separated at 80 volts for 60 minutes. The GeneRuler™ 1 kb Plus DNA Ladder (Thermo Fischer Scientific, Darmstadt, Germany) was used as marker. The DNA was stained in an ethidium bromide bath (1% solution on 500 mL) for 15 minutes. The gel was documented in a UV transilluminator (SynGene Genius, Bio Imaging System).


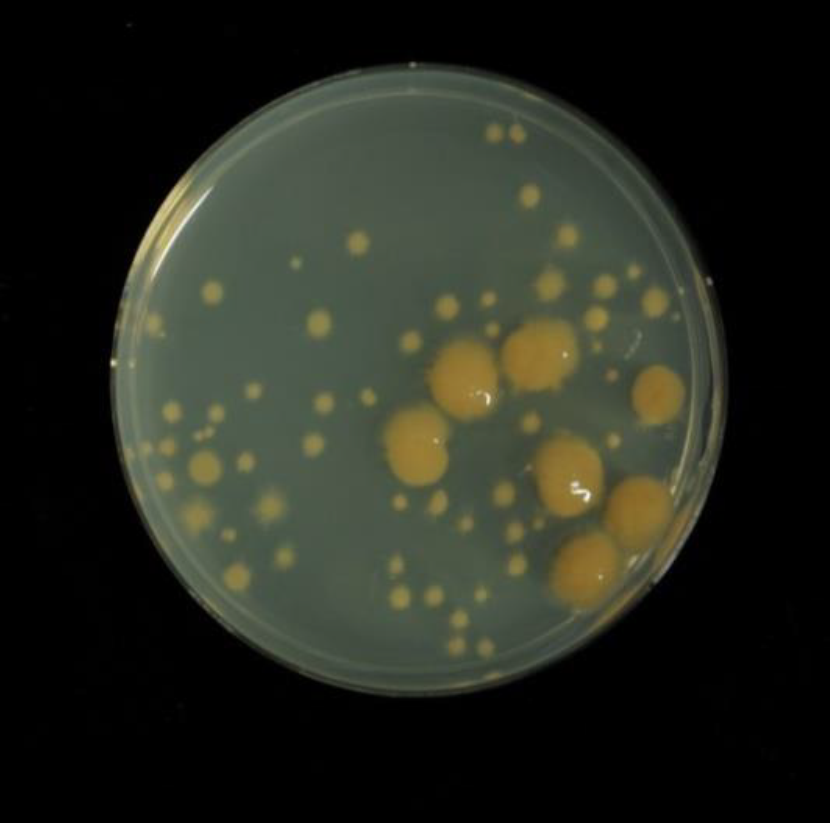


**Supplementary Figure 2:** *Aspergillus sydowii* Fsh102 grown in 50 mL mineral medium with 1% xylan after 3 days at 28°C.


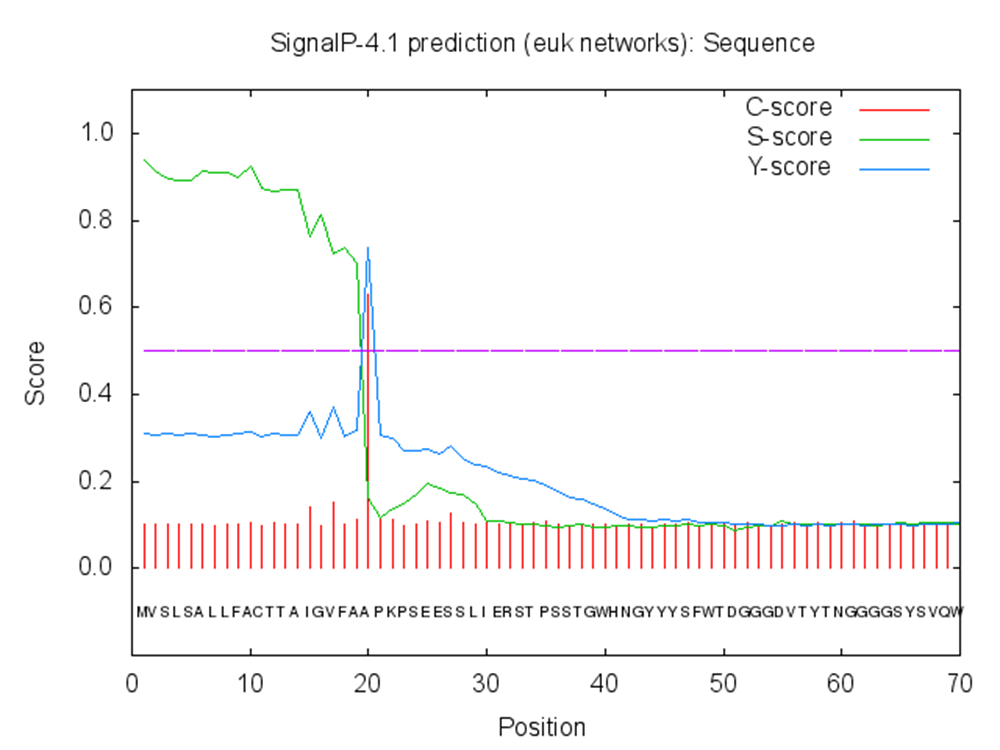


**Supplementary Figure 3:** Signal peptide prediction of xylanase I from *Aspergillus sydowii* Fsh102 by SignalP4.1 (Nielsen, 2017). The red line above the purple dotted line indicated a clear signal peptide cleavage site. The green line represents a potential signal peptide.


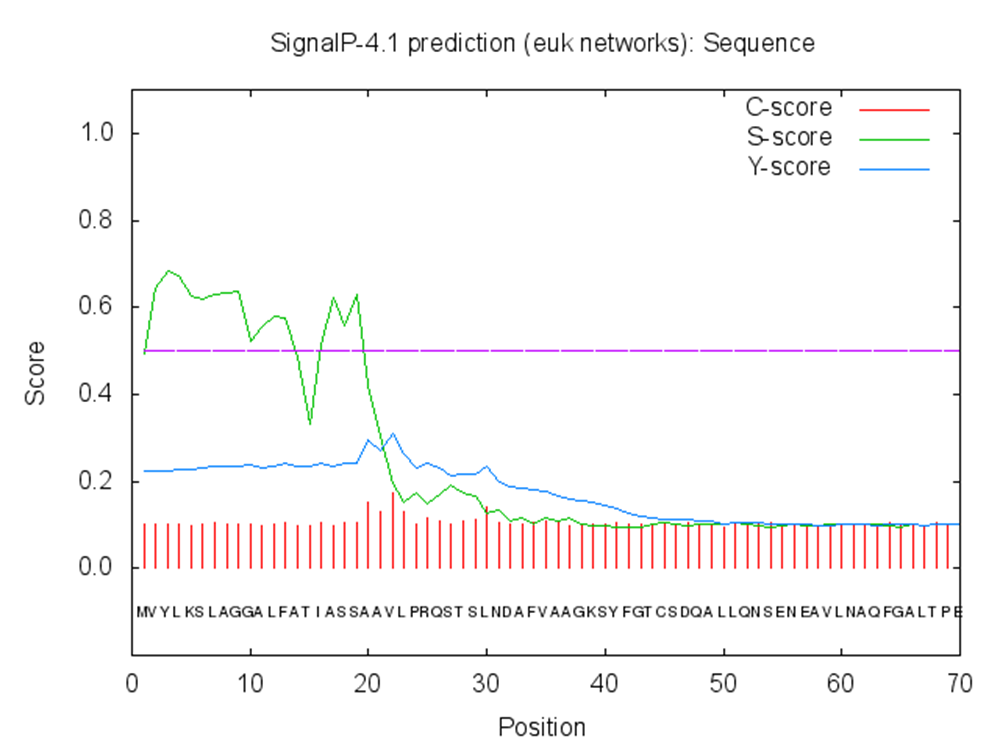


**Supplementary Figure 4:** Signal peptide prediction of xylanase II from *Aspergillus sydowii* Fsh102 SignalP4.1 (Nielsen, 2017). The green line represents a potential signal peptide.


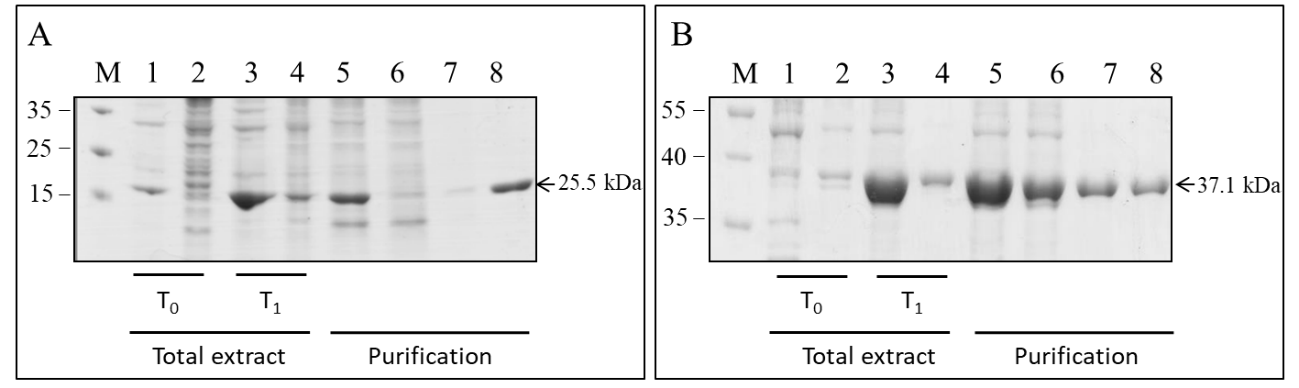


**Supplementary Figure 5:** Electrophoretic separation of different protein fractions with the heterologously expressed (A) xylanase I and (B) xylanase II. The enzymes were purified on a Ni-NTA column and separated by 12.5% (w/v) SDS-PAGE. Coomassie Brilliant Blue was used for staining. The calculated molecular weight of xylanase I without signal peptide is 25.5 kDa. The calculated molecular weight of xylanase II without signal peptide is 37.1 kDa.

Lane 1: IF T0, Lane 2: SF T0 at 22 °C, Lane 3: IF of expression T1, Lane 4: SF of expression T1, Lane 5: extract of SF at T1, Lane 6: flowthrough of purification, Lane 7: flow of washing step, Lane 8: elution 1 of protein. M: Page Ruler Plus Prestained Protein Ladder (Thermo Fisher Scientific, Darmstadt, Germany). T0 = before induction, T1 = at 22 °C after 16 h after induction with IPTG, IF = insoluble fraction, soluble fraction = SF. The molecular weights of the standard are given in kDa on the right


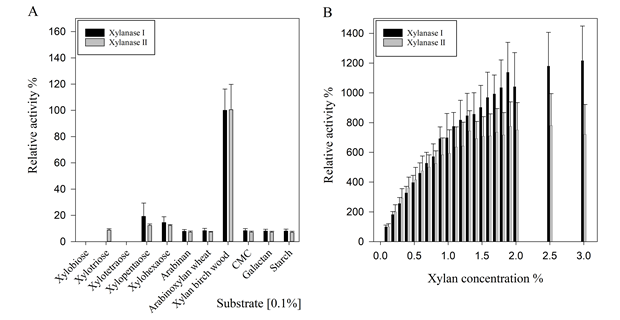


**Supplementary Figure 6:** The substrate spectrum (A) and the activity at increasing xylan concentration (B) of the xylanase II (black) and the xylanase II (grey). The relative activities (%) were determined by DNS method. The measurements were performed in triplicates. (A) All activities of xylanase I and xylanase II were each measured on the activity at pH 4.8 and related to the natural substrate xylan from beech wood = 100%. The specific activity was determined with xylobiose, xylotriose, xylotetraose, xylopentose, xylohexose, arabinan, arabinoxylan, CMC, galactan and starch (B) The specific activity was determined at xylan concentrations between 0.1% and 3.0% (w/v) and related to the activity at 0.1% xylan = 100%.


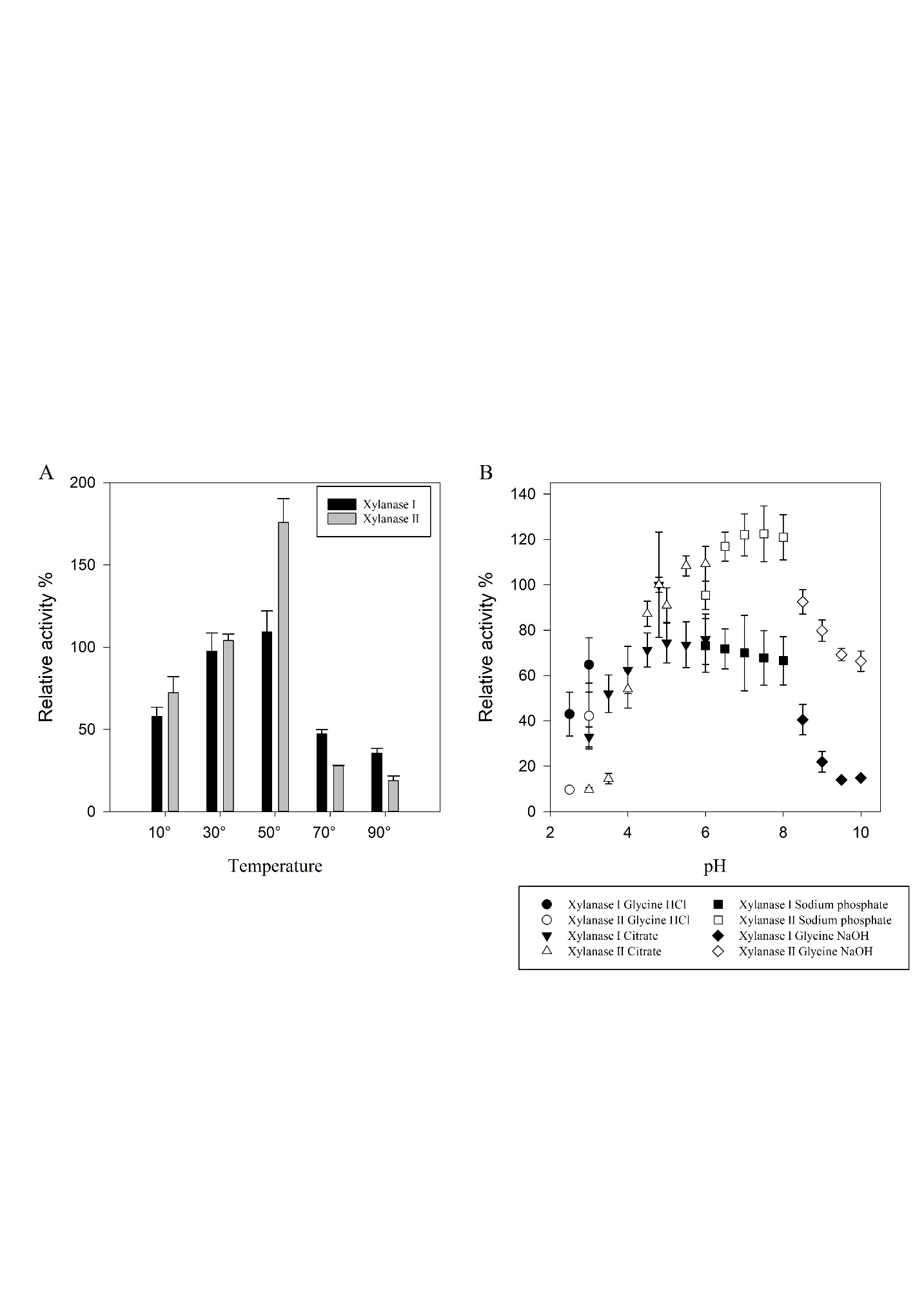
**Supplementary Figure 7:** Effects of the activity by temperature (A) and pH-value (B) of the xylanase I (black) and xylanase II (grey or white). (A) The activities were determined using 10 °C, 30 °C, 50 °C, 70 °C and 90 °C. 100% are set for 30°C for both enzymes. (B) The dependency of the activity and the pH-values between 2.5 and 10.0, as well as the buffer salts were determined. For the pH-values from 2.5 to 3.0 glycine HCL buffer, from 3.0 to 6.0 citrate buffer, from 6.0 to 8.0 sodium phosphate buffer and from 8.5 to 10.0 glycine NaOH buffer were used. 100% are set for pH 4.8 for both enzymes.

**
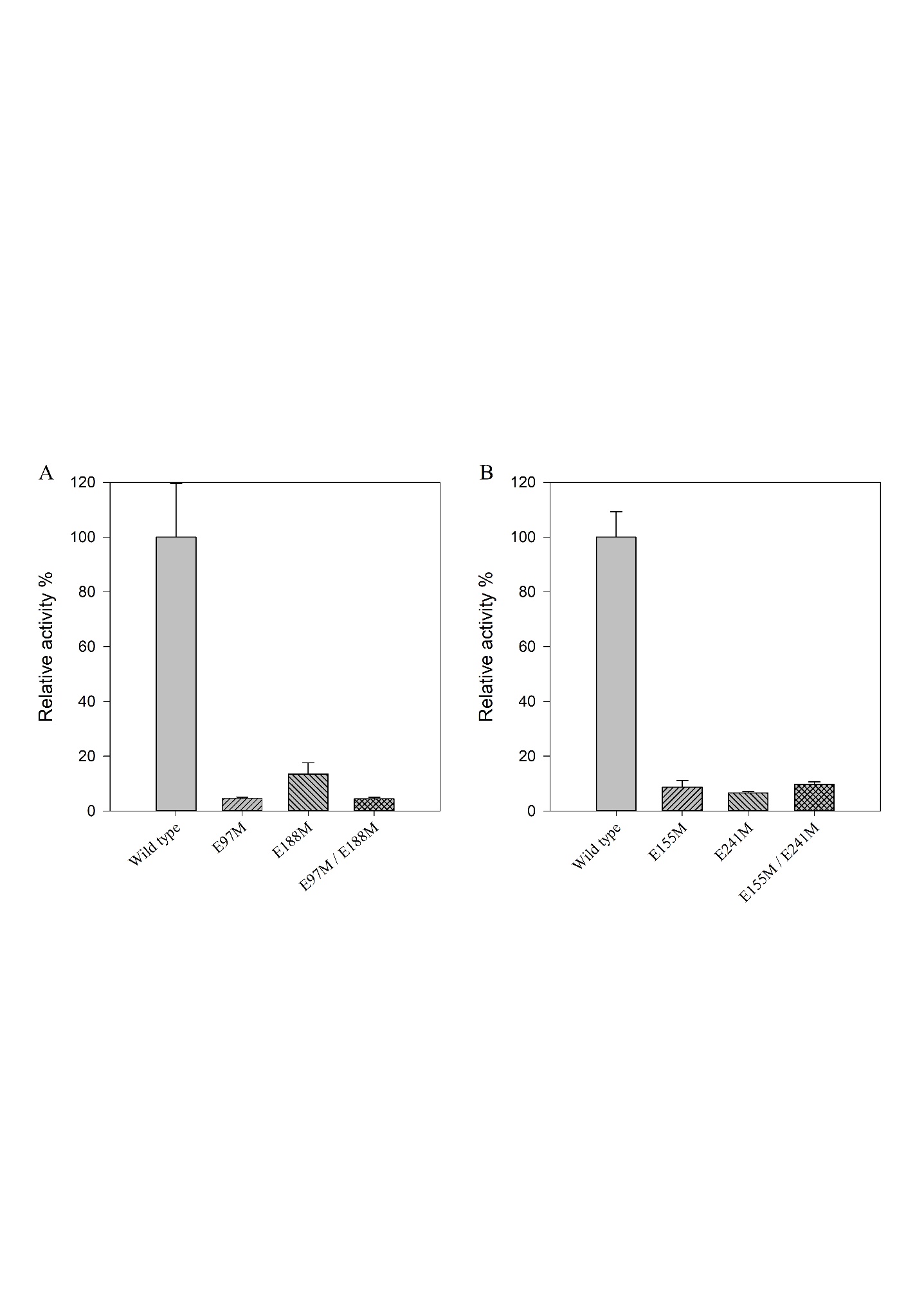
**

**Supplementary Figure 8:** Relative activity (%) of single and double mutants of xylanase I (A) and xylanase II (B), measured with the DNS method (Miller, 1959) and related to the activity of the non-mutated enzymes.


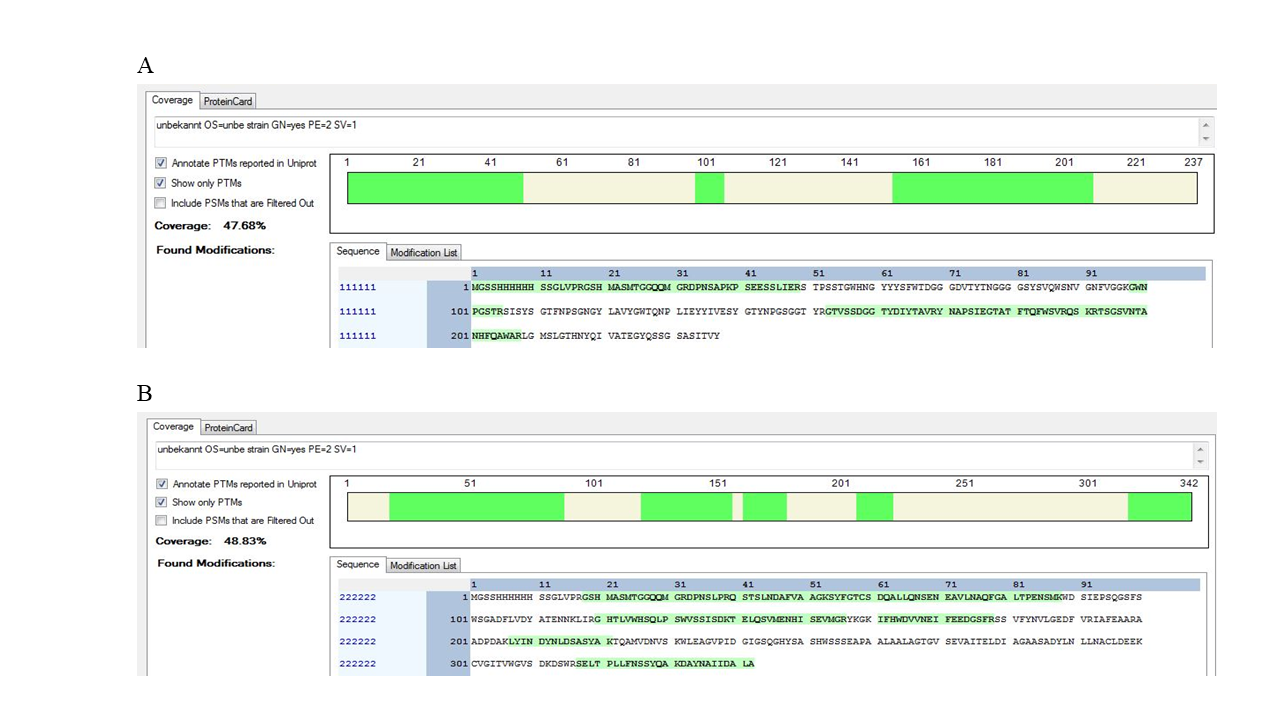


**Supplementary Figure 9:** Verification of A) xylanase I and B) xylanase II by liquid chromatography with coupled mass spectrometry (LC/MS-MS). A) The coverage % of the amino acid sequence of purified xylanase I is 47.68%. B) The coverage % of the amino acid sequence of xylanase II is 48.83%. The green areas indicate the amino acids found, the yellow areas indicate the amino acids which were not found.


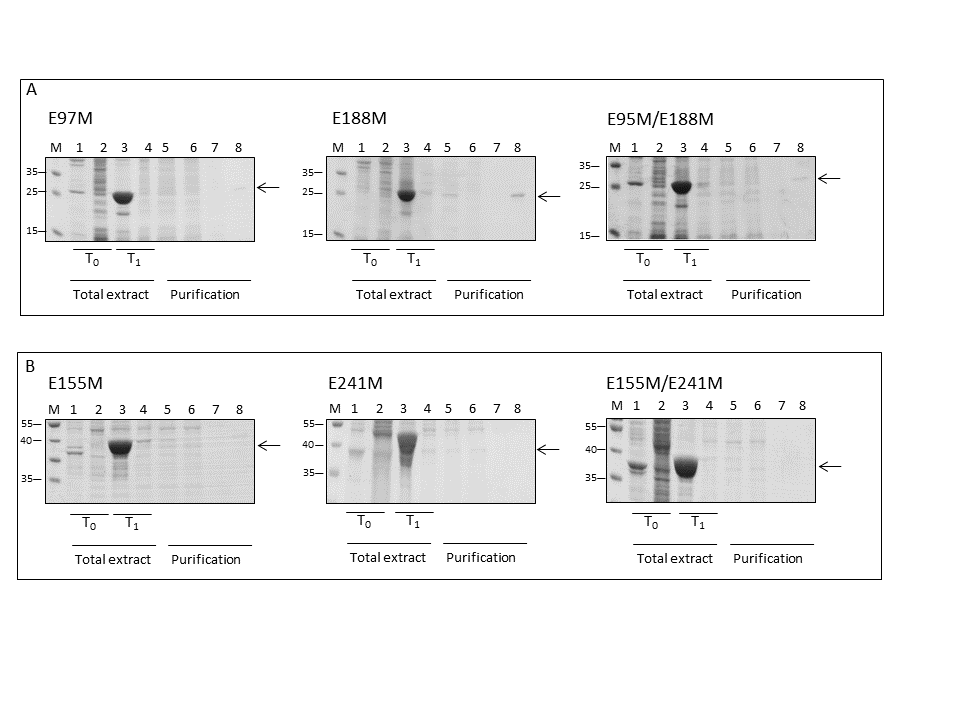


**Supplementary Figure 10:** Electrophoretic separation of different protein fractions with the heterologously expressed (A) xylanase I mutants E97M, E188M and E95M/E188M and (B) xylanase II mutants E155M, E241M and E155M/E241M. The enzymes were purified on a Ni-NTA column and separated by 12.5% (w/v) SDS-PAGE. Coomassie Brilliant Blue was used for staining. The calculated molecular weight of xylanase I mutants without signal peptide is 25.5 kDa indicated with an arrow. The calculated molecular weight of xylanase II mutants without signal peptide is 37.1 kDa indicated with an arror.

Lane 1: IF T0, Lane 2: SF T0 at 22°C, Lane 3: IF of expression T1, Lane 4: SF of expression T1, Lane 5: extract of SF at T1, Lane 6: flowthrough of purification, Lane 7: flow of washing step, Lane 8: elution 1 of protein. M: Page Ruler Plus Prestained Protein Ladder (Thermo Fisher Scientific, Darmstadt, Germany). T0 = before induction, T1 = at 22°C after 16 h after induction with IPTG, IF=insoluble fraction, SF=soluble fraction. The molecular weights of the standard are given in kDa on the left.


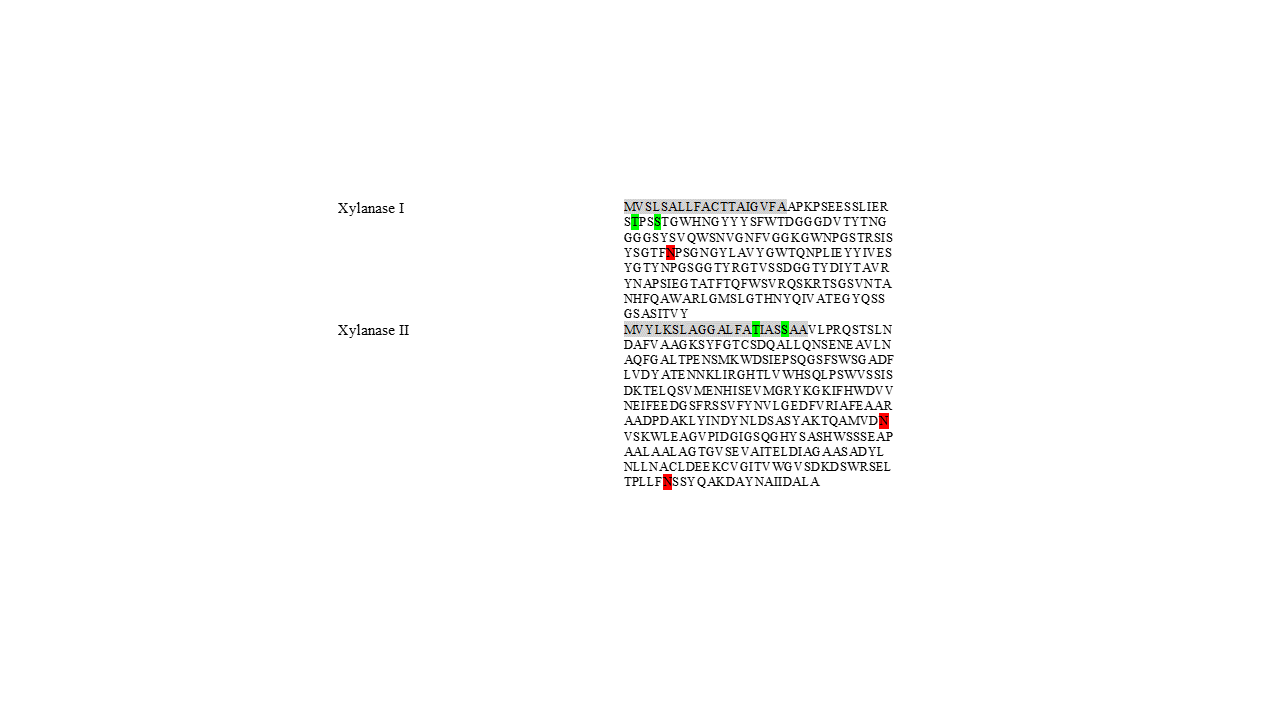


**Supplementary Figure 11:** Amino acid sequences of xylanase I and xylanase II. The grey-shaded amino acids mark the putative signal peptide. The amino acids marked in red show the N-glycosylation sites. The green marked amino acids show the O-glycosylation sites.


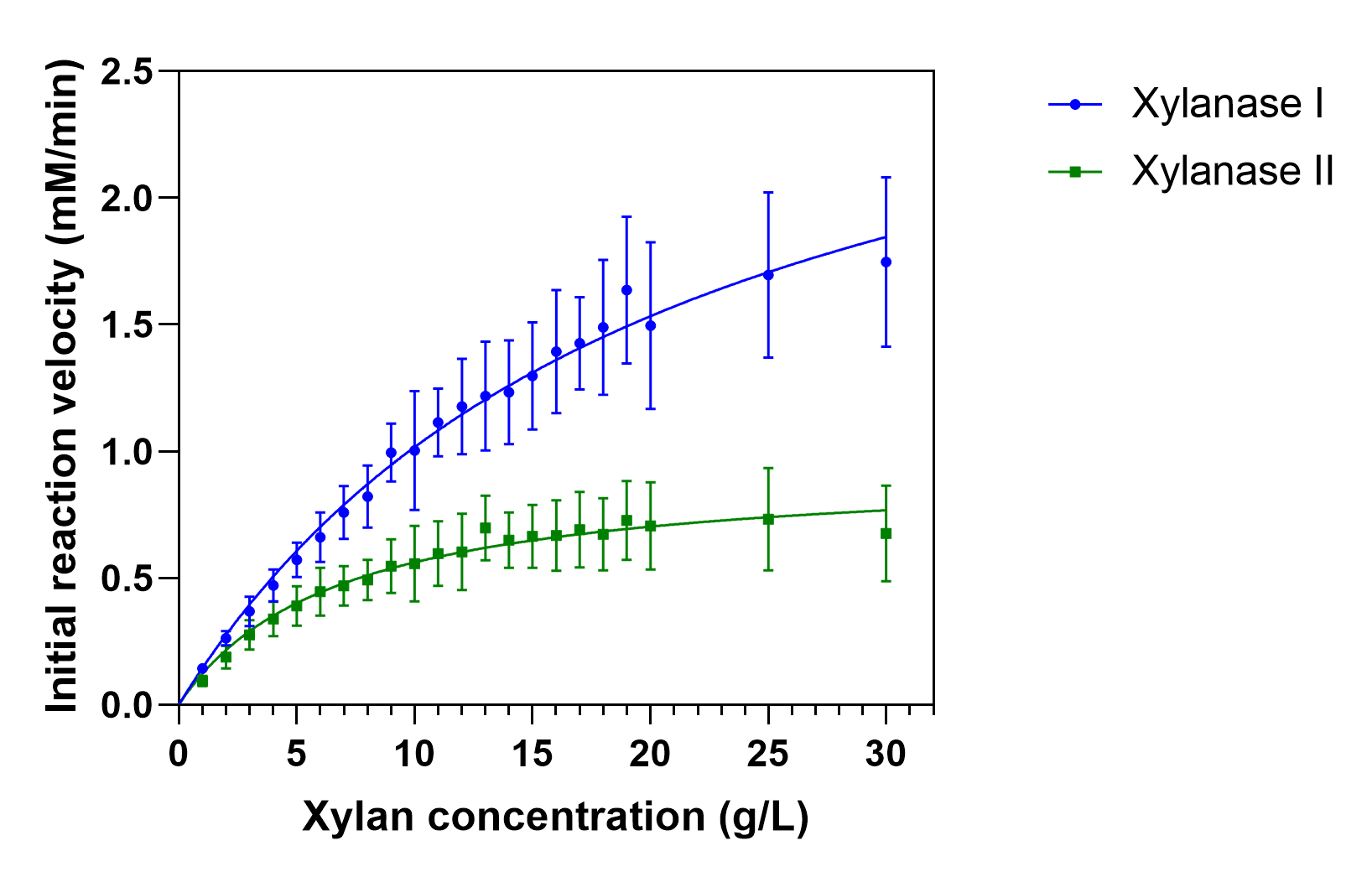


**Supplementary Figure 12:** Michaelis-Menten plots for the determination of K_M_ (g/L) and V_max_ (mM/min) values of the xylanases. The initial reaction velocities (determined from the reducing sugars produced and expressed as equivalent xylose) were obtained in the standard assay mixture at pH 4.8 and 30 °C with 1 µg xylanase I or xylanase II respectively.

## Supplementary Tables

**Supplementary Table 1**: Activity of the supernatant of 24 fungal isolates induced with xylan. The reactions were performed as describe before (Brandt *et al*., 2018). The values are the mean of three independent cultivations. The Fsh102 is marked red.

| Name | DSMZ ID | Unit | | Unit/mL | | Unit/mg | | phylogenetic assignment |
| --- | --- | --- | --- | --- | --- | --- | --- | --- |
|  |  | average | standard deviation | average | standard deviation | average | standard deviation |  |
| FL100 | 106490 | 0.39623 | 0.02022 | 3.96233 | 0.20220 | 1.02780 | 0.05025 | *Penicillium citrinum* strain |
| FR1 | 106296 | 0.55062 | 0.01736 | 5.50616 | 0.17361 | 0.97223 | 0.02354 | *Aspergillus oryzae* strain |
| Fsh17 | 106723 | 0.36607 | 0.01322 | 3.66074 | 0.13219 | 0.73625 | 0.02625 | *Aspergillus flavus* strain |
| Fsh200 | 107515 | 0.42275 | 0.01446 | 4.22751 | 0.14465 | 0.71210 | 0.02494 | *Aspergillus sydowii* strain |
| FL10 | 106248 | 0.38186 | 0.00949 | 3.81860 | 0.09489 | 0.69272 | 0.01853 | *Penicillium citrinum* strain |
| FW30 | 106733 | 0.33488 | 0.01430 | 3.34882 | 0.14303 | 0.65430 | 0.02795 | *Talaromyces marneffei* strain |
| FW49 | 106685 | 0.32018 | 0.01778 | 3.20181 | 0.17778 | 0.64163 | 0.03696 | *Calocybe indica* strain |
| Fsh21 |  | 0.32551 | 0.00774 | 3.25507 | 0.07742 | 0.63973 | 0.01849 | *Calocybe indica* strain |
| Fsh201 | 107514 | 0.44746 | 0.01761 | 4.47460 | 0.17609 | 0.63421 | 0.02613 | *Penicillium citrinum* strain |
| Fi10 | 106453 | 0.38091 | 0.01124 | 3.80913 | 0.11237 | 0.63220 | 0.01842 | Aspergillus flavus strain |
| Fsh102 | 105790 | 0.29776 | 0.00570 | 2.97762 | 0.05698 | 0.61409 | 0.01188 | *Aspergillus sydowii* strain |
| FW33 | 106711 | 0.27904 | 0.00831 | 2.79042 | 0.08312 | 0.58338 | 0.01616 | *Trichoderma* sp. |
| Fi21 | 106463 | 0.33474 | 0.01451 | 3.34739 | 0.14509 | 0.58127 | 0.02307 | *Aspergillus flavus* strain |
| SF19.1 | 106380 | 0.29685 | 0.01815 | 2.96855 | 0.18152 | 0.57307 | 0.03482 | *Aspergillus flavus* strain |
| Fsh20 | 106730 | 0.31460 | 0.00902 | 3.14605 | 0.09023 | 0.55464 | 0.01640 | *Aspergillus flavus* strain |
| FL6 | 106243 | 0.29928 | 0.00956 | 2.99281 | 0.09555 | 0.52238 | 0.01883 | *Aspergillus terreus* strain |
| FW24 | 106709 | 0.14875 | 0.00867 | 1.48752 | 0.08668 | 0.45922 | 0.02511 | *Fusarium fujikuroi* strain |
| FF1 | 104516 | 0.25169 | 0.00912 | 2.51686 | 0.09123 | 0.45214 | 0.01613 | *Aspergillus nomius* strain |
| FW36 | 106736 | 0.26506 | 0.01700 | 2.65056 | 0.16995 | 0.44000 | 0.03086 | *Aspergillus* sp. |
| Fsh101 | 107513 | 0.32267 | 0.00832 | 3.22665 | 0.08319 | 0.43506 | 0.01145 | *Aspergillus sydowii* strain |
| Fi4 | 106445 | 0.24749 | 0.01354 | 2.47489 | 0.13539 | 0.41382 | 0.02251 | *Talaromyces aurantiacus* strain |
| SF25 | 106387 | 0.14765 | 0.00807 | 1.47647 | 0.08070 | 0.41032 | 0.02399 | *Talaromyces flavus* strain |
| FR27 | 106324 | 0.08698 | 0.00121 | 0.86984 | 0.01213 | 0.30023 | 0.00443 | *Trichoderma asperellum* strain |
| SF31 | 106393 | 0.14610 | 0.00990 | 1.46098 | 0.09900 | 0.23093 | 0.01557 | *Aspergillus terreus* strain |

**Supplementary Table 2**: Proteins of the fungal isolate Fsh102 induced with xylan. The enzymes were identified from the zymogram with 0.5% xylan by liquid chromatography with coupled mass spectrometry. The annotation, description, coverage (%), number of peptides (# peptides) and molecular weight in kDa (MW [kDa]) were compared with the automated translation of the genome of the fungal isolate *Aspergillus sydowii* CBS 593.65. The repeat column displays if the proteins were found in other bands again and the frequency. The putative xylanases are marked in bold.

| Number | Annotation | Description | Coverage [%] | # Peptides | MW [kDa] | Band | Repeat |
| --- | --- | --- | --- | --- | --- | --- | --- |
| 1 | Actin | Aspsy1 51743\|fgenesh1_kg.19_#_75_#_Locus237v1rpkm814.15 | 9.07 | 2 | 41.63 | 1 | - |
| 2 | Heat shock protein 90 | Aspsy1 61916\|fgenesh1_pm.15_#_251 | 2.00 | 1 | 79.52 | 1 | - |
| 3 | Heat shock protein 70 | Aspsy1 33431\|fgenesh1_pg.9_#_39 | 2.49 | 1 | 69.76 | 1 | - |
| 4 | Polyubiquitin | Aspsy1 53937\|fgenesh1_pm.1_#_980 | 11.80 | 1 | 34.32 | 1 | - |
| 5 | Putative ubiquitin-40S ribosomal protein | Aspsy1 87723\|gm1.4406_g | 5.84 | 1 | 17.62 | 1 | - |
| 6 | Histone | Aspsy1 118465\|gw1.3.1613.1 | 9.78 | 1 | 10.19 | 1 | - |
| 7 | Hypothetical protein | Aspsy1 42610\|fgenesh1_kg.3_#_604_#_Locus415v1rpkm469.49 | 4.36 | 3 | 85.73 | 1 | - |
| 8 | **Putative Exo-1,4-beta-xylosidase** | Aspsy1 84747\|gm1.1430_g | 4.59 | 2 | 84.49 | 1 | - |
| 9 | Hypothetical protein | Aspsy1 28486\|fgenesh1_pg.2_#_1138 | 1.98 | 1 | 115.18 | 1 | - |
| 10 | Hypothetical protein | Aspsy1 203580\|estExt_Genewise1Plus.C_6_t10196 | 1.49 | 1 | 84.35 | 1 | - |
| 11 | Hypothetical protein | Aspsy1 152814\|e_gw1.6.52.1 | 3.11 | 1 | 57.80 | 1 | - |
|  |  |  |  |  |  |  |  |
| 12 | Actin | Aspsy1 51743\|fgenesh1_kg.19_#_75_#_Locus237v1rpkm814.15 | 11.22 | 3 | 41.63 | 2 | same as 1 |
| 13 | Heat shock protein 90 | Aspsy1 61916\|fgenesh1_pm.15_#_251 | 2.00 | 1 | 79.52 | 2 | same as 2 |
| 14 | Putative Heat shock protein 70 chaperone | Aspsy1 39214\|fgenesh1_kg.1_#_1450_#_Locus444v1rpkm444.85 | 1.78 | 1 | 73.81 | 2 | - |
| 15 | Putative Glucan endo-1,3-β-glucosidase | Aspsy1 156803\|e_gw1.9.955.1 | 4.38 | 2 | 47.72 | 2 | - |
|  |  |  |  |  |  |  |  |
| 16 | **Putative Endo-1,4-**β**-xylanase** | Aspsy1 63902\|estExt_fgenesh1_pg.C_1_t20379 | 13.41 | 3 | 35.43 | 3 | - |
| 17 | Putative Glucan Endo-1,3-β-glucosidase | Aspsy1 156803\|e_gw1.9.955.1 | 2.51 | 1 | 47.72 | 3 | same as 15 |
| 18 | Hypothetical protein | Aspsy1 55895\|fgenesh1_pm.2_#_1217 | 3.68 | 1 | 44.03 | 3 | - |
|  |  |  |  |  |  |  |  |
| 19 | **Putative Endo-1,4-**β**-xylanase** | Aspsy1 168372\|estExt_Genewise1.C_1_t60491 | 15.00 | 2 | 23.52 | 4 | - |
| 20 | Putative aldehyde dehydrogenase | Aspsy1 36479\|fgenesh1_pg.16_#_248 | 3.98 | 1 | 54.46 | 4 | - |
| 21 | Putative transcription factor | Aspsy1 31982\|fgenesh1_pg.6_#_90 | 1.96 | 1 | 105.71 | 4 | - |
| 22 | Mediator of RNA polymerase II | Aspsy1 576851\|CE361813_11644 | 4.95 | 1 | 34.85 | 4 | - |
| 23 | Hypothetical protein | Aspsy1 203580\|estExt_Genewise1Plus.C_6_t10196 | 1.49 | 1 | 84.35 | 4 | same as 10 |
| 24 | Hypothetical protein | Aspsy1 55895\|fgenesh1_pm.2_#_1217 | 3.68 | 1 | 44.03 | 4 | same as 18 |

**Supplementary Table 3**: Proteins of the fungal isolate Fsh102 induced with xylan. The enzymes were identified after the zymogram with 0.5% xylan by liquid chromatography coupled with mass spectrometry. The proteins were identified by comparison to the automated translation of the genome of the fungal isolate *A. sydowii* CBS 593.65.

| **#** | **Protein identifier** | **Protein sequence** |
| --- | --- | --- |
| 1 | >jgi\|Aspsy1\|33431\|fgenesh1_pg.9_#_39 | MAPAVGIDLGTTYSCVGVFRDDRIDIIANDQGNRTTPSFVAFTDTERLIGDAAKNQVAMNPHNTVFDAKRLIGRRFADAEVQADMKHWPFKIIDKTGKPVIQVEFKGETKEFTPEEISSMVLTKMRETAEAFLGGTVNNAVVTVPAYFNDSQRQATKDSGLIAGLNVLRIINEPTAAAIAYGLDKKVEGERNVLIFDLGGGTFDVSLLTIEEGIFEVKATAGDTHLGGEDFDNRLVNHFVNDFKRKNKKDLSTNARALRRLRTACERAKRTLSSAAQTSIEIDSLFEGIDFYTSITRARFEELCQDLFRSTMEPVERVLRDAKIDKSSVHEIVLVGGSTRIPKIQRLVSDYFNKEPNKSINPDEAVAYGAAVQAAILSGDTSSKSTNEILLLDVAPLSVGIETAGGVMTALVKRNTTIPTKKSETFSTYSDNQPGVLIQVYEGERARTKDNNLLGKFELTGIPPAPRGVPQIEVTFDLDANGIMNVSAIEKGTGKTNKITITNDKGRLSKEDIERMLADAEKYKAEDEAEAARIQAKNGLESYAYSLKNTIGEGQLQISEDDKKKVSDKVDEVISWLDTNQTAEKDEYESQQKELEGVANPIISAAYAAAGGAPGGAPPGAGAAPGGGATRADGEVEENDDLD* |
| 2 | >Aspsy1 53937\|fgenesh1_pm.1_#_980 | MQIFVKTLTGKTITLEVESSDTIDNVKTKIQDKEGIPPDQQRLIFAGKQLEDGRTLSDYNIQKESTLHLVLRLRGGMQIFVKTLTGKTITLEVESSDTIDNVKTKIQDKEGIPPDQQRLIFAGKQLEDGRTLSDYNIQKESTLHLVLRLRGGMQIFVKTLTGKTITLEVESSDTIDNVKTKIQDKEGIPPDQQRLIFAGKQLEDGRTLSDYNIQKESTLHLVLRLRGGMQIFVKTLTGKTITLEVESSDTIDNVKTKIQDKEGIPPDQQRLIFAGKQLEDGRTLSDYNIQKESTLHLVLRLRGGN* |
| 3 | >jgi\|Aspsy1\|87723\|gm1.4406_g | MQIFVKTLTGKTITLEVESSDTIDNVKSKIQDKEGIPPDQQRLIFAGKQLEDGRTLSDYNIQKESTLHLVLRLRGGGKKRKKKVYTTPKKIKHKRKKTKLAVLKYYKVDGDGKIERLRRECPAPECGAGIFMAAMQNRQYCGKCHLTYVFDESK* |
| 4 | >jgi\|Aspsy1\|118465\|gw1.3.1613.1 | RKETYSSYIYKVLKQVHPDTGISTRAMSILNSFVNDIFERVATEASKLAAYNKKSTISSREIQTSVRLILPGELAKHAVSEGTKAVTKYSSS* |
| 5 | >jgi\|Aspsy1\|42610\|fgenesh1_kg.3_#_604_#_Locus415v1rpkm469.49 | MVAPTIVLQIGCFFGFLICDTPRGVWPEGRPTVSPWPSSSSSPGISTSTGGTATSGETSVPVTVPTVAPTVTVSTPGTTLTTLPSTDSTTSGPISSTTVSSSTVSSSTSTWTTSISSPSSTPSTTSTMSVSTSAVPAATGTLAANILILARDSTQADVASSGLNGYGIPYTTLLVPQAGVELPALNSSSGGNFGGIVVAGEVSYDYGSDSWRSALTDDQWNQLYAYQLAYGVRMVQYDVFPGPKFGASSVGDGGCCADGVEQLVYFSDTSDFPTAGLRTGSNAGVSTEGLWHYPASVSDTNTTKKIASFAAGGGVSGESAAAVINNFDGRQQMVFFIGFDTVWSQTSNYLQHAWITWITRGLYAGHRRVNLNTQIDDMFLETDIYEPAGQIFRIGTADMDGITSWLPTIRAKLNAGSTYFVEIGHNGNGNIEAAVESAGENTCSGGAIEYDSPPDTELEFQKPLGTGTDLWPSSPTNYTWTTTCTNSDSLLVWFQEHLNDYAYISHTFTHLEQNNATYSDIYKEISFNQAWLRQVGFAAAERFTSNGIIPPAITGLHNGDALRAWSDNGITNCVGDNTRPALLNTENAMWPYFTKVSSDGFDGIQVNPRWATRIYYNCDTPACTTQEWIDTSAGAGTFTDLLATERAETLRHFFGLHRDPFMFHQANLRNTGIDPITVNGEEAQISIFQAWVETVVAEFTRLVNWPVVSTTHQEMSDYFLQRYRRDQCGYSLSYVLDNNSITGVTLSANGNSCDAAIPVTFPDAPSDTQGFSTEQLGSDPVTVWAQLSGSPVTFSLASPIAL* |
| 6 | >jgi\|Aspsy1\|84747\|gm1.1430_g | MKPLSLAAWAALLPAAAAQANESFVDYNTEANPNLFPQCLAHLNHSFPSCSSGPLANTPVCDRSLDPMTRAKGLVSLFTLDELLNNTGNTNLGVPRLNLPNYQVWGEALHGVGRANFQDSGKFSWATSFPMPINTMSALNRTLIQHIGSIVSTQLRAFSNAGHGGVDVYSPNINTFRHPVWGRGQETPGEDAFLAAVFGYEYITALQGGVDPETIKIIATAKHYAGYDIESWNGHSRLGNDMQITQQELSEYYMPPFMVAAMDAKVRSVMCSYNAVNGVPGCANIFFLQTLLRDTFGFVEEGYVSGDCGAVYNVWNPHGYAGNESAASAASVLAGTDIDCGTSYQWYLDEAAGDALVARTEIERGVVRLYASLVAAGYFDGEDAKYRDISWEDVLETDAWDVAYQAAVESIVLLKNDGALPLKDSVKSVAVVGPWANVTEELQGNYFGPAPYLISPLDAFKESDFDVHYAKGADLNSNSTAGFEAALKAARKADVVVFAGGIDNTIEAEAMDRENITWPGNQLDLINQLSKVGKPLVVLQMGGGQVDSSSLKNNDNVNALIWGGYPGQSGGHALFDIITGAKAPAGRLVATQYPANYAMDFPATDMNLRPSGDNPGQTYMWYTGEPVYEFGHGLFYTTFEESTKSTTAGPFNIQEILAETHPGHARVEQKTLLNFTAAVKNTGDVKSDYTALVFANTTAGPAPYPTKWVVGFDRLGGLESGDSQTLTVPVSIESVARTDEKGNRVLYPGSYELALNNERSVVVSFELKGDKAVLLSWPEDTTSP* |
| 7 | >jgi\|Aspsy1\|28486\|fgenesh1_pg.2_#_1138 | MDEPAGETGLQADESTHGRVDNGILLDPVTPTEYIRLLEDRLYKLESEFYDLEGVVRSDHGPAQSDETTTDSEIDSVSSVQCDKQSEESPNPPQSAKPIIPSLSKVSWFGFHNLLEGDDVFAIDVCSVKTAGCYNDDASSRKFRDRGASLNYKQGVQMFLDFKVPPRGADVVNSIRINSMAILTLLHDIDPRVPIDGSPLRLDPPFLSLIHYHDQVKQILDRTRSAVXTSDTDTERHRAEELLRHMDPYMHFMDTEVLPKYRRLGLQEVNTVNAKVRFSELPYLFRRDDDIYQPAGTDALSEDSLGHQEAWRILPERISSFYQNNTEKFCCFDVICYHIAFDGRSYVPIRKCLTIVEFEGERDVTSLPMYPLRFMPNHREIRTELRNRGARFLEYINSKQVSYQGWALQHLDGHGNDRTYISSNLVVDFAETSRAHPDWADYVRKSDWKNDFEENPFASPEDGQSTLWELSRTTNKLEVKTCKTESVYDLSLPHEQTDTCLQKHRLILEFNKRQEEPTAIGQLQDELEIQEDELVLLPKRLFAYALQDRKFVMLDIANARSIAQTGDPFKELIISRSHKDMISSLVHAHFEKKQMEQIHGFYHISQDIIHNKGRGLVILLHGVPGVGKSSTAEAVAQRWKRPLLAITCGDLGLEAADVEHSLKEVFRLAQLWDCVLLLDEADVFLSQRSVSDLQRNSLVSVFLRVLEYYSGILFLTTNRVGHIDEAFMSRIHISLYYPRLDLDQTIDIWRMNIDRLQSIEKKRSEATGMPALTIAADEILEYAKKHFHARGPDRAWNGRQIRNAFQTAAALARYESDTSLNKTLENPHHVLARHFKTVARAGRGFEDYLRETRGKSDSDLAYDTKTRADHIIENRPQKPPIVSEAASGFGQSKYQSTYLNPSQDNHPSPNIRAGGYQTAPQNYILSSSGQYTPNAYSQGRASSPQQLYNAATNPFLSTMNAGNPRHTTPSRSMTPQPPMEPRGGNFSYECVQNPSLSLGQYHIPQDDDEFSD* |
| 8 | >jgi\|Aspsy1\|152814\|e_gw1.6.52.1 | MAANPNFTWTSTTPGRWERDVDEAEQFYTSLAKAYEGTGRNFFAMTGFISLSIPLPESTPQAEAEKNLESALRKAWTHLRYDHPTIASRVVYDADQKKCKKVYHTFSPSYTVQDWLDETFIVVDEGISGLEWCNSDPPVPDFPTLFFIKTGLHDGRIGGDLVLRSQHDIIDGMGTLLLFHNFLSHTARAHVHQGQNASPGYNKYEPPHFGDEYKNLSPPLCIAAQIPGELDEAHKHRMSQIQKYNATLRNNNVELACPPFKREHDGGPGKHQRVAIRLSQEETKSVVAACRELRLSITHAYHAAIPLVVRDLQEKRESERQVRYLNYSLINERGNCVEPYSTSSHPAAVYHSVSGKGLAVDLTVPALETRTRTSTSTKTKYANVASTIRKFYLDTRADKDHIFLVPSYWQMGTIPYPADGKKQPVPSRNETPSVSISSMGILDKVIGHRYGEIEVEEPWVTGEELGTGLGLFLGTWKGRVTLSAAYNDAWHDKEEVVEFINKCNEVTLSGLDLQP* |
| 9 | >jgi\|Aspsy1 51743\|fgenesh1_kg.19_#_75_#_Locus237v1rpkm814.15 | MEEEVAALVIDNGSGMCKAGFAGDDAPRAVFPSIVGRPRHHGIMIGMGQKDSYVGDEAQSKRGILTLRYPIEHGVVTNWDDMEKIWHHTFYNELRVAPEEHPVLLTEAPINPKSNREKMTQIVFETFNAPAFYVSIQAVLSLYASGRTTGIVLDSGDGVTHVVPIYEGFALPHAISRVDMAGRDLTDYLMKILAERGYTFSTTAEREIVRDIKEKLCYVALDFEQEIQTASQSSTLEKSYELPDGQVITIGNERFRAPEALFQPSVLGLESGGIHVTTFNSIMKCDVDVRKDLYGNIVMSGGTTMYPGISDRMQKEITALAPSSMKVKIIAPPERKYSVWIGGSILASLSTFQQMWISKQEYDESGPSIVHRKCF* |
| 10 | >jgi\|Aspsy1\|61916\|fgenesh1_pm.15_#_251 | MSETFEFQAEISQLLSLIINTVYSNKEIFLREIISNSSDALDKIRYESLSDPSKLDSGKDLRIDIIPDAENKTLTIRDTGIGMTKADLINNLGTIARSGTKQFMEALSAGADISMIGQFGVGFYSAYLVADRVTVISKHNDDEQYIWESAAGGTFTLTQDTEGEALGRGTKMIFHLKDEQAEYLQESRIKEVVRKHSEFISYPIYLHVLKETEKEVPDEEAESKEEGDDEKKPKIEEVDEDEEKKEKKTKTVKESKIEEEELNKTKPIWTRNPADITEEEYAAFYKSLSNDWEDHLAVKHFSVEGQLEFRAILYVPKRAPFDLFETKKTKNNIKLYVRRVFITDDATDLIPEWLGFVKGVVDSEDLPLNLSRETLQQNKIMKVIKKNIVKKTLELFTEIAEDREQFDKFYSAFAKNIKLGIHEDAQNRNTLAKLLRYQSTKSGDETTSLTDYVTRMKEHQKQIYYITGESIKAVAKSPFLDSLKQKDFEVLFLVDPIDEYAFTQLKEFDGKKLVDITKDFELEETEEEKAEREKEEKEFEDLAKALKNILGDKVEKVVVSHKLIGSPCAIRTGQFGWSANMERIMKAQALRDTSMSSYMSSKKTFEISPKSSIVKELKKKVEADGESDRTVKSITQLLYETSLLVSGFTIDEPASFAERIHKLVSLGLNIDEEAEAEPATTEEAPAAATTGESAMEEVD* |
| 11 | >jgi\|Aspsy1\|39352\|fgenesh1_kg.1_#_1450_#_Locus444v1rpkm444.85 | MSRQSGRSAAKPFAAWTTVFYLLLVFIAPLAFFGTAHAQEDEVQENYGKVIGIDLGTTYSCVGVMREGRVEIMVNDQGNRITPSYVAFTDDERLVGDAAKNQYAANPKRTIFDIKRLIGRKYDESDVQRDIKNFPFKVVNNDGKPNVRVDVNQSPKTLSPEEVSAMVLGKMKEVAEGYLGEKVTHAVVTVPAYFNDAQRQATKDAGTIAGLNILRVVNEPTAAAIAYGLDKGEKEGERQVIVYDLGGGTFDVSLLSIDSGVFEVLATAGDTHLGGEDFDHRVMDYFVKQYNKKNNVDVTKDLKAMGKLKREVEKAKRTLSSQKSTRIEIEAFHKGEDFSETLTRAKFEELNMDLFKKTMKPVEQVLKDAKVSKAEVDDIVLVGGSTRIPKVQAILEEYFGKQASKGINPDEAVAFGAAVQGGVLSNEEGTGGVVLMDVNPLTLGIETTGGVMTKLIPRNTVIPTRKSQIFSTAADNQPTVLIQVFEGERSLTKHNNVLGKFELTDIPPAPRGVPQIEVAFDLDANGILKVSASDKGTGKAETITIKNDKGRLTPEDIERMVAEAEQFAEEDRAIKTKIEARNGLENYAFSLKNQVSNEEGLGGQIDEDDKQTILDAVKEVHDWLDENGASATTEDFEEQKEQLSNVAYPITSKLYGSAPPTEEDDDDYGHDEL* |
| 12 | >jgi\|Aspsy1\|156803\|e_gw1.9.955.1 | MFTKTQILAFALSLASAEAASKGFNYGNNKPDGSLKAQADFQSEFHTAKNLEGTSGFNSARLYTMIQGEGNSPIEAIPAAIAEETTLLLGLWASGGNMDNEIAALKSAINQYGDAFTKLVVGISVGSEDLYRNSEIGVQANAGIGVEPEELVGYVQRVREAVSGTSLSDAPIGHVDTWNSWTNGSNNAVIEAVDWLGFDGYPYFQSEMDNGIDNAKNLWDEAVGKTKNVANGKPVWITEGGWPVSGDQQNLAVASIENAKRFWDEVGCPLFDEVNTWWYILQDAAGDSVPNPSFGIVGNSLTTKPLYDLSCKANFGSGGSSSGGSSSGAASSGAASTISASPSGSGFAQGSSTVSPSPLPSSSGSGLGSGGSLGSSGSFPGSGFGSSSSMVISPSGSATAIPSSSAGAGSSGSTGVGAGTGASSGNGASGSGSGSGTGTSDQSTSSTSPSPAEFTGAGTRLSGSILGAAVLVAALAVAL* |
| 13 | >jgi\|Aspsy1\|63902\|estExt_fgenesh1_pg.C_1_t20379_Xylanase II | MVYLKSLAGGALFATIASSAAVLPRQSTSLNDAFVAAGKSYFGTCSDQALLQNSENEAVLNAQFGALTPENSMKWDSIEPSQGSFSWSGADFLVDYATENNKLIRGHTLVWHSQLPSWVSSISDKTELQSVMENHISEVMGRYKGKIFHWDVVNEIFEEDGSFRSSVFYNVLGEDFVRIAFEAARAADPDAKLYINDYNLDSASYAKTQAMVDNVSKWLEAGVPIDGIGSQGHYSASHWSSSEAPAALAALAGTGVSEVAITELDIAGAASADYLNLLNACLDEEKCVGITVWGVSDKDSWRSELTPLLFNSSYQAKDAYNAIIDALA* |
| 14 | >jgi\|Aspsy1\|156803\|e_gw1.9.955.1 | MFTKTQILAFALSLASAEAASKGFNYGNNKPDGSLKAQADFQSEFHTAKNLEGTSGFNSARLYTMIQGEGNSPIEAIPAAIAEETTLLLGLWASGGNMDNEIAALKSAINQYGDAFTKLVVGISVGSEDLYRNSEIGVQANAGIGVEPEELVGYVQRVREAVSGTSLSDAPIGHVDTWNSWTNGSNNAVIEAVDWLGFDGYPYFQSEMDNGIDNAKNLWDEAVGKTKNVANGKPVWITEGGWPVSGDQQNLAVASIENAKRFWDEVGCPLFDEVNTWWYILQDAAGDSVPNPSFGIVGNSLTTKPLYDLSCKANFGSGGSSSGGSSSGAASSGAASTISASPSGSGFAQGSSTVSPSPLPSSSGSGLGSGGSLGSSGSFPGSGFGSSSSMVISPSGSATAIPSSSAGAGSSGSTGVGAGTGASSGNGASGSGSGSGTGTSDQSTSSTSPSPAEFTGAGTRLSGSILGAAVLVAALAVAL* |
| 15 | >jgi\|Aspsy1\|55895\|fgenesh1_pm.2_#_1217 | MRSLFGFLATLATLTVGTTALEASVISFGSNPQGQGSKSSVTSSATLQRLLELRSKSFTASELEGSDESSIDFLGRLAGTPGQLFGAPVADGDLDTVTVILEGLDKEAESSIQNEYESELVVSRFATDAARDDFIDYLLENRLDGIVSPESKHCSFYNHSKHRDGYFQTNEDFLRFCLPRNFGSQKFNRGFDSQLLSQADTGESWLNARKGTIVVHISFEASSTYIHSLRSLFFELHSHSLNGKTATAVVLPSSKPRPKSPYSKRSPHVTKAGTSTSLARDTQSIVFEQRADIPLSLAPVCYASNSSCNEATNTCSGHGACYKKSGDCYACRCHETYVKTESGVEQKVRWGGAACQKRDISSPFFLITGVTITIMAVVSAAVGMIFRVGNTELPGVIGAGVGPSRTQK* |
| 16 | >Aspsy1 168372\|estExt_Genewise1.C_1_t60491_Xyl1 | MVSLSALLFACTTAIGVFAAPKPSEESSLIERSTPSSTGWHNGYYYSFWTDGGGDVTYTNGGGGSYSVQWSNVGNFVGGKGWNPGSTRSISYSGTFNPSGNGYLAVYGWTQNPLIEYYIVESYGTYNPGSGGTYRGTVSSDGGTYDIYTAVRYNAPSIEGTATFTQFWSVRQSKRTSGSVNTANHFQAWARLGMSLGTHNYQIVATEGYQSSGSASITVY* |
| 17 | >jgi\|Aspsy1\|36479\|fgenesh1_pg.16_#_248 | MSDLFVEITAPNGRKYTQPTGLFINNEFVASSGQTITSIDPATDKPIATVHAASVDDVDRAVKAAKAALVHPSWKNLPGTERGQLMGRLADLMEQNKELFATIDAWDNGKPYHVALNEDLVEAIGTIRYYSGWADKTFGQTISTTPEKFAYTIRQPVGVVGQIIPWNYPLSMACWKLGPALACGNTVVLKPAEQTPLSILIFAKLFKEAGFPPGVVNFVNGYGREAGAALAGHPLVDKIAFTGSTMTAREIMKLAAGTLKNITLETGGKSPLLVFPDADLEQAVKWSHFGIMSNQGQICTATSRIYVHQDIFQTFLARFKEAVETTSKIGDQWDENTFQGPQVTRAQYDRILSYIEAAKKDGVKVVTGGAAHAPADAKNKNGYFVQPTVFTGESDSYAIVREEVFGPVVVILPFSSEEDAIKRANNTTYGLGAAVFTRDLERAHRVAAEIEAGMVWVNSSQDCDPRVPFGGVKQSGIGRELGEAGLEAYTQVKAVHVNMGNKL* |
| 18 | >jgi\|Aspsy1\|31982\|fgenesh1_pg.6_#_90 | MRSSIACARCRRSKIKCVNSGIDTTCRACDSSGRECVYPTPAIGVGAAAKRDIAALADGEDRNGDWDSPKRQRSRKTVGLSSAARDASKASLEALDSSVLTIKVWEAVFDLFQAHYATILPFLHPASFMGQIRQLSGNNHTSLPTSSNNASSTAIPNPQEPPRDQAPNPPSTPPNPLIPLGVLALTARFHPQLAAYHSPSSPGNPPNPLVASEFYATALRSRLAGVDGANLAVPDLTRVQALLMLALHEWGMCRGKSAWLYVGMAIRMSQAMGLPFELENDVFSRDAPRDSALKTEADMFGITSRPEQKEQNQSDEVIAQETKRRTFWACFILDRSLSSGKYRPRMIRVKELDIQLPSENAFAFGERVRTSRLTDPVGRRPQSFSSASQGAQQIPSLRHSIGGYSEGKMPQNSADNHPWSPVSGRKDSTEEEIDRWEIGAEESVLSRAVRITRVWGSIAKWSCAGGRRNEQLPPWHPDSRFYRLRTSLAEFRDALSRNLQYSPRNTDTHIMYKNNLLHPYTFIHLVYFLSVIVLHRAYIPFLPVRCAEPVGPLDEPVDKTGMPEGFWRDSARELFAAARQLMDLAVTCQERGVLVENPLVGFAIYNAAFVGVYATHFPQMDLDGVLAPVQRGDPQGQLQSRKALAVLREMRPRLKMAAGWFRTLNRLHSYFSKVKRDFRRNSRRGDMLPPDAIDPHNVNGIRPVREGGSGGGLEEFKLLEKLFLEFGSIDDQLTDGSGNEEDGDRATNVSDTGSNHIRSDPGDLGEGPLEGAGGRRESWVPVNSPGLPLPSLDANGERRPSLPLPPGRSLQSQSPFSLPSLQHHPDGSQYNNSSPTLPSLAPSSAYGGLPNTSTPASSARLQPINSWLNSRSQPPPASYSQSLPPISAAAPAHGLPMLPPPGSIGHPGASPPATLDGLETFNSLWSTSLGGDDVLAFLEGSECTQYPSAASEVGVPAGWLSTVWTEFAQ* |
| 19 | >Aspsy1 576851\|CE361813_11644 | MAGTQDPSALEEILWRSPSHVQMMGGYLHSNNILFYFAESPFFDATSNNASLAIQANYNEAFRHFVETREAFEGRLKTMQGLEFIVGYDPLQAAAGSNAQFAHEPSNVWVIRKQMRRKRSGFEDEVVALATFFVVGDCIYMAPSAASILGNRILSAVTSLSSLMKTASTLPNFTPSHGHTYLPPAPKSTDAGQLGVQTQTSKENTPMPDDATNKTQSFTGSQADASSSVSDMMTLAESFNLLARYGDEFMDETPLVGEPGSFILSRAGDTDRGATSKQQQLPSTAAPGRIGTPQTKADTPGKSSDKGSTVEEPKRKKKSKPGS* |
| 20 | >Aspsy1 203580\|estExt_Genewise1Plus.C_6_t10196 | MSTTVEAPRQYGQPSRKGKRAWRKNVDVSEVQEGLRLLKDEEIKGGVLAEKPSEELFVIDKKGSSEIRDAYRKQHKKPLKADEILAQRSAIAAVDTRKRGSSNVTDGVIEPKTKKHKSDWVSRKEWKRLKQVAKEGNPMDKPSDGGFYDPWADEADPTPLDDPQFDYLEKPKPKVAPETLKRPPISLAADGKAVPSVRAPTAGTSYNPTFEDWDALLREQGQRAVEEEKKRLEEERKEQERQRLIDEAKNDDGEAKSDDESAWEGFESEYEKPEWLNKKRPERKTKTQRNKIKRRKEAERQAKWEEQKKKKEAQVAEAKAISERFKLQQLERQERDSDADESSDEGDDTDLRRKPLGGKIRAPEKPTEVVLPDELQDSLRLLKPEGNLLDDRFRTLIVQGKLESRKPVSQPRKAKREVTEKWTYKDFKHPAHIGMELKSLSSNWKRLQETLKKDVSTGPTPKRKRAEPESQNDQVKKRKTVTEAGKAAKRISQTAYATKKRNRMSQAPSNAVGKETQSISRKNSTAAFPKDNEGRSSTVEIGKYIAVDCEMVGIGPNPDNDSVLARISIVNYNGDQVYDSYVRPKEMVTDWRTHVSGITPKHMVEARSLELVQKEVAEILDGRILVGHALRNDLDALLLSHPKRDIRDTSKHAPYRKIAGGGSPRLKILASEFLGLKIQEGAHSSVEDAKATMLLYRRDKDEFEREHLKKWPVRVAPENNENGGDEKKKKKKKKKTRKR* |
| 21 | >jgi\|Aspsy1\|55895\|fgenesh1_pm.2_#_1217 | MRSLFGFLATLATLTVGTTALEASVISFGSNPQGQGSKSSVTSSATLQRLLELRSKSFTASELEGSDESSIDFLGRLAGTPGQLFGAPVADGDLDTVTVILEGLDKEAESSIQNEYESELVVSRFATDAARDDFIDYLLENRLDGIVSPESKHCSFYNHSKHRDGYFQTNEDFLRFCLPRNFGSQKFNRGFDSQLLSQADTGESWLNARKGTIVVHISFEASSTYIHSLRSLFFELHSHSLNGKTATAVVLPSSKPRPKSPYSKRSPHVTKAGTSTSLARDTQSIVFEQRADIPLSLAPVCYASNSSCNEATNTCSGHGACYKKSGDCYACRCHETYVKTESGVEQKVRWGGAACQKRDISSPFFLITGVTITIMAVVSAAVGMIFRVGNTELPGVIGAGVGPSRTQK* |

**Supplementary Table 4:** DNA sequences of potential xylanases of the fungal isolate Fsh102 induced with xylan. The enzymes were identified after the zymogram with 0.5% xylan by liquid chromatography coupled with mass spectrometry.

| **#** | **Identifier** | **DNA sequence** |
| --- | --- | --- |
| 1 | >Xyl1 CDS\|jgi\|Aspsy1\|168510\|estExt_Genewise1.C_1_t60491 | ATGGTTTCGCTCTCTGCCCTCCTCTTCGCTTGCACCACTGCAATCGGTGTCTTCGCCGCCCCTAAACCATCTGAAGAGTCAAGCTTGATTGAGCGCTCCACCCCAAGCTCCACCGGCTGGCACAATGGCTACTATTACTCCTTCTGGACCGACGGCGGCGGCGATGTGACCTACACCAACGGCGGCGGCGGATCGTATTCAGTGCAGTGGTCTAATGTTGGAAACTTTGTCGGTGGAAAGGGTTGGAATCCTGGAAGTACACGATCCATAAGCTACAGCGGAACCTTCAACCCCAGCGGTAACGGCTACCTTGCCGTATACGGCTGGACCCAGAACCCTCTAATCGAGTACTACATTGTTGAATCATACGGCACCTACAACCCCGGCAGTGGGGGGACGTATCGCGGAACAGTGAGCTCTGATGGCGGGACATACGACATCTACACTGCGGTGCGGTACAATGCGCCCTCGATTGAAGGGACGGCCACGTTTACGCAGTTCTGGTCGGTGCGCCAGTCGAAGCGTACTTCGGGGAGCGTCAATACTGCTAATCATTTCCAGGCGTGGGCGAGGCTGGGCATGAGTCTGGGGACGCATAATTATCAGATTGTGGCCACGGAGGGGTATCAGAGTAGTGGGTCGGCTTCGATTACTGTTTACTAG |
| 2 | >Xylanase II CDS\|jgi\|Aspsy1\|64040\|estExt_fgenesh1_pg.C_1_t20379 | ATGGTCTACCTCAAATCCCTCGCCGGAGGTGCGCTCTTCGCCACAATCGCCTCGTCGGCCGCCGTTCTCCCTCGCCAGTCGACAAGCCTCAACGATGCCTTTGTCGCCGCGGGCAAGTCGTACTTTGGCACCTGCTCCGACCAGGCCCTGCTGCAGAACAGCGAGAACGAGGCCGTGCTGAACGCGCAGTTTGGGGCGCTGACGCCGGAGAATAGCATGAAGTGGGATTCTATTGAGCCATCCCAGGGCAGTTTTAGCTGGAGCGGTGCGGACTTCTTGGTCGACTACGCGACTGAGAACAACAAGTTGATTCGCGGACACACCCTTGTGTGGCACTCGCAGCTCCCGTCGTGGGTATCCTCCATCAGCGACAAGACTGAACTGCAGAGCGTCATGGAGAACCACATCAGCGAGGTCATGGGTCGGTATAAGGGAAAGATCTTCCACTGGGACGTCGTCAACGAAATCTTCGAGGAAGACGGCTCGTTCCGTAGCAGCGTCTTCTACAACGTCCTCGGCGAGGACTTTGTCAGGATTGCGTTTGAGGCTGCGCGTGCCGCGGACCCGGATGCGAAGCTGTATATAAATGACTATAACCTCGACTCCGCAAGCTACGCCAAGACGCAGGCGATGGTCGACAATGTGTCCAAGTGGCTGGAGGCTGGTGTTCCGATTGATGGAATTGGCTCCCAGGGTCATTATAGTGCGAGCCACTGGTCGAGCTCTGAAGCTCCAGCAGCTCTGGCCGCTCTCGCTGGCACCGGGGTGTCTGAAGTCGCCATCACCGAGCTCGACATCGCGGGCGCCGCATCAGCCGACTACCTGAACTTGCTGAATGCCTGTCTGGACGAGGAGAAATGCGTGGGCATCACCGTCTGGGGCGTGTCGGACAAGGACTCGTGGCGGTCGGAGCTCACCCCCTTGCTGTTTAACAGCAGCTACCAGGCCAAGGACGCCTACAACGCGATTATCGATGCACTGGCGTAG |
| 3 | >jgi\|Aspsy1\|84747\|gm1.1430_g | ATGAAGCCCCTTTCTCTCGCCGCGTGGGCAGCCCTGCTCCCAGCAGCAGCCGCCCAGGCAAACGAGAGCTTCGTCGACTACAACACCGAAGCCAACCCTAATCTCTTCCCCCAATGCCTCGCACATCTCAACCACTCTTTCCCCTCCTGCAGCAGCGGGCCTCTCGCCAATACCCCCGTATGCGATCGCAGCCTTGACCCGATGACACGCGCCAAAGGCCTCGTCTCCCTCTTCACCCTCGACGAACTCCTCAACAACACTGGAAACACCAACCTGGGCGTGCCCCGCCTCAATCTCCCCAACTACCAAGTCTGGGGCGAAGCCCTGCACGGCGTCGGCCGCGCTAATTTCCAAGATTCCGGCAAATTCAGCTGGGCCACCTCCTTCCCCATGCCCATCAACACCATGAGCGCCCTGAACCGCACTCTGATCCAGCATATCGGGTCGATTGTCTCGACGCAGCTGCGCGCTTTCAGCAACGCCGGCCACGGCGGCGTGGACGTCTACTCCCCCAATATCAATACCTTCCGCCACCCCGTCTGGGGCCGCGGGCAGGAGACACCCGGTGAGGACGCGTTCCTTGCGGCTGTGTTCGGGTACGAGTATATCACGGCTCTACAGGGCGGTGTTGATCCCGAGACGATCAAGATTATCGCCACGGCGAAGCATTACGCCGGGTATGACATTGAGAGCTGGAATGGGCATTCAAGGCTGGGTAATGATATGCAGATCACGCAGCAGGAGCTATCGGAGTACTATATGCCGCCGTTTATGGTTGCGGCTATGGATGCGAAGGTTCGCAGTGTGATGTGCTCGTATAACGCCGTGAACGGGGTTCCGGGCTGCGCCAACATATTCTTCTTGCAGACGCTGCTGAGGGATACCTTTGGGTTTGTGGAGGAAGGGTATGTCTCTGGGGATTGTGGTGCTGTGTATAATGTGTGGAATCCGCATGGGTATGCGGGTAATGAGTCTGCGGCCAGTGCGGCGTCTGTGCTTGCCGGGACGGATATTGACTGCGGGACCTCGTATCAGTGGTATCTGGACGAGGCGGCTGGGGATGCGCTTGTTGCGAGGACCGAAATTGAGAGGGGTGTTGTGAGGCTTTATGCGTCGCTTGTGGCGGCTGGGTACTTTGACGGTGAGGATGCGAAGTATAGGGATATTTCGTGGGAGGATGTGCTGGAGACGGATGCGTGGGATGTTGCGTACCAGGCTGCGGTGGAGAGTATCGTCCTGCTGAAGAACGATGGTGCTTTGCCACTGAAGGACAGTGTCAAGAGTGTTGCTGTTGTTGGTCCCTGGGCGAATGTGACCGAGGAGCTGCAGGGCAATTACTTTGGACCTGCGCCGTATCTGATCAGTCCGTTGGATGCGTTCAAGGAGTCTGACTTTGATGTCCACTATGCCAAGGGTGCTGATCTCAACTCGAACTCAACTGCTGGGTTCGAGGCTGCACTCAAGGCTGCGAGGAAGGCCGATGTGGTTGTCTTTGCTGGTGGTATTGACAACACCATCGAGGCAGAGGCCATGGACCGAGAGAACATCACCTGGCCGGGCAACCAGCTGGACCTGATTAACCAGCTTAGCAAGGTTGGAAAGCCGTTGGTTGTCTTGCAGATGGGCGGAGGACAGGTCGATTCGTCCTCGCTGAAGAACAACGACAACGTCAATGCCCTGATCTGGGGAGGATACCCCGGCCAGTCTGGCGGCCATGCCTTGTTCGATATCATCACTGGAGCAAAGGCACCTGCAGGCCGTCTCGTTGCCACACAGTATCCGGCCAACTATGCCATGGACTTCCCAGCAACTGACATGAACCTCCGTCCCAGCGGGGATAACCCTGGCCAGACTTATATGTGGTACACCGGCGAGCCAGTGTACGAGTTCGGCCATGGTCTGTTCTATACCACGTTCGAGGAGTCTACCAAGAGCACCACTGCAGGACCGTTTAATATCCAGGAGATCCTGGCTGAGACGCATCCCGGCCATGCACGCGTTGAGCAGAAGACCCTGCTCAACTTTACCGCAGCCGTCAAGAACACTGGAGATGTCAAGTCCGACTACACAGCCCTGGTCTTTGCCAACACCACCGCTGGCCCGGCGCCGTATCCGACCAAGTGGGTTGTCGGGTTTGACCGTCTGGGAGGACTGGAGTCTGGCGATTCGCAGACCCTGACCGTGCCTGTCTCGATTGAGAGCGTTGCACGCACGGACGAGAAGGGCAACAGGGTGCTCTACCCAGGATCGTACGAGCTAGCCCTGAACAACGAGAGATCGGTCGTTGTCTCGTTTGAGCTGAAGGGTGACAAGGCAGTTCTTCTAAGCTGGCCAGAGGACACCACGAGCCCGTAA |

**Supplementary Table 5:** Used primer for ITS sequencing and cloning of xylanase I and xylanase II.

| **Primer** | **Sequence (5‘ – 3‘)** | **Scope of application** | **Reference** |
| --- | --- | --- | --- |
| ITS1 [F] | TCCGTAGGTGAACCTGCGG | PCR primer to amplify the ITS region | White *et al.*, 1990 |
| ITS4 [R] | TCCTCCGCTTATTGATATGC | PCR primer to amplify the ITS region | White *et al.*, 1990 |
| pET28b [F] | CCCGCGAAATTAATACGACTCAC | PCR primer for verification  of cloning in pET28b | this work |
| pET28b [R] | CTAGTTATTGCTCAGCGGT | PCR primer for verification  of cloning in pET28b | this work |
| X102_1_G [F] | GGTCGGGATCCGAATTCCATGGTTTCGCTCTCTGCCC | PCR primer of the gene Xylanase I | this work |
| X102_1_G [R] | AGTGCGGCCGCAAGCTTCTAGTAAACAGTAATCGAAGCCG | PCR primer of the gene Xylanase I | this work |
| X102_1_V [F] | CGGCTTCGATTACTGTTTACTAGAAGCTTGCGGCCGCACT | PCR primer of the gene Xylanase I | this work |
| X102_1_V [R] | GGGCAGAGAGCGAAACCATGGAATTCGGATCCCGACC | PCR primer of the gene Xylanase I | this work |
| X102_2_G [F] | GGTCGGGATCCGAATTCCATGGTCTACCTCAAATCCCTC | PCR primer of the gene Xylanase II | this work |
| X102_2_G [R] | AGTGCGGCCGCAAGCTTCTACGCCAGTGCATCGATAA | PCR primer of the gene Xylanase II | this work |
| X102_2_V [F] | TTATCGATGCACTGGCGTAGAAGCTTGCGGCCGCACT | PCR primer of the gene Xylanase II | this work |
| X102_2_V [R] | GAGGGATTTGAGGTAGACCATGGAATTCGGATCCCGACC | PCR primer of the gene Xylanase II | this work |
| Xylanase I_w/o_SP [F] | AAATGGGTCGGGATCCGAATTCCGCCCCTAAACCATCTGAAGAGTC | PCR primer to remove the signal peptides from Xylanase I | this work |
| Xylanase I_w/o_SP [R] | TCAGATGGTTTAGGGGCGGAATTCGGATCCCGACCCATTT | PCR primer to remove the signal peptides from Xylanase I | this work |
| Xylanase II_w/o_SP [F] | AAATGGGTCGGGATCCGAATTCCCTCCCTCGCCAGTCGACAAG | PCR primer to remove the signal peptides from Xylanase II | this work |
| Xylanase II_w/o_SP [R] | CTTGTCGACTGGCGAGGGAGGGAATTCGGATCCCGACCCATTT | PCR primer to remove the signal peptides from Xylanase II | this work |
| X1_E97M [F] | CAGAACCCTCTAATCATGTACTACATTGTTGAATC | PCR primer to create the single mutant from Xylanase I | this work |
| X1_E97M [R] | GATTCAACAATGTAGTACATGATTAGAGGGTTCTG | PCR primer to create the single mutant from Xylanase I | this work |
| X1_E188M [F] | GTGGCCACCATGGGGTATCAG | PCR primer to create the single mutant from Xylanase I | this work |
| X1_E188M [R] | CTGATACCCCATCGTGGCCAC | PCR primer to create the single mutant from Xylanase I | this work |
| X2_E241M [F] | GTCGCCATCACCATGCTCGACATCGCG | PCR primer to create the single mutant from Xylanase II | this work |
| X2_E241M [R] | CGCGATGTCGAGCATGGTGATGGCGAC | PCR primer to create the single mutant from Xylanase II | this work |
| X2_E155M [F] | GGGACGTCGTCAACATGATCTTCGAGGAAG | PCR primer to create the single mutant from Xylanase II | this work |
| X2_E155M [R] | CTTCCTCGAAGATCATGTTGACGACGTCCC | PCR primer to create the single mutant from Xylanase II | this work |
| Xylanase II_w/o_SP [R] | CTTGTCGACTGGCGAGGGAGGGAATTCGGATCCCGACCCATTT | PCR primer to remove the signal peptides from Xylanase II | this work |
| X1_E97M [F] | CAGAACCCTCTAATCATGTACTACATTGTTGAATC | PCR primer to create the single mutant from Xylanase I | this work |
| X1_E97M [R] | GATTCAACAATGTAGTACATGATTAGAGGGTTCTG | PCR primer to create the single mutant from Xylanase I | this work |
| X1_E188M [F] | GTGGCCACCATGGGGTATCAG | PCR primer to create the single mutant from Xylanase I | this work |
| X1_E188M [R] | CTGATACCCCATCGTGGCCAC | PCR primer to create the single mutant from Xylanase I | this work |
| X2_E241M [F] | GTCGCCATCACCATGCTCGACATCGCG | PCR primer to create the single mutant from Xylanase II | this work |
| X2_E241M [R] | CGCGATGTCGAGCATGGTGATGGCGAC | PCR primer to create the single mutant from Xylanase II | this work |
| X2_E155M [F] | GGGACGTCGTCAACATGATCTTCGAGGAAG | PCR primer to create the single mutant from Xylanase II | this work |
| X2_E155M [R] | CTTCCTCGAAGATCATGTTGACGACGTCCC | PCR primer to create the single mutant from Xylanase II | this work |

**Supplementary Table 6:** CAZyme analysis of the fungal isolates Fsh102 and different other fungal species in particular *Aspergillus* species. The coded regions (CDS) were compared with the CAZyme database (Cantarel *et al*. 2009; Lombard *et al*. 2014).

| CAZyme Family | *Aspergillus_Sydowii*_FSH102 | *Aspergillus_Sydowii*_JGI | *Aspergillus_aculeatus*_ATCC16872 | *Aspergillus_carbonarius*_ITEM5010 | *Aspergillus_clavatus*_NRRL1 | *Aspergillus_flavus*_NRRL3357 | *Aspergillus_oryzae*_3_042 | *Aspergillus_terreus*_NIH2624 | *Aspergillus_niger*_CBS_513_88 | *Fusarium_oxysporum* | *Fusarium_graminearum*_PH_1 | *Magnaporthe_grisea*_DS9461 |
| --- | --- | --- | --- | --- | --- | --- | --- | --- | --- | --- | --- | --- |
| GH1 | 4 | 4 | 3 | 3 | 4 | 3 | 2 | 3 | 3 | 6 | 3 | 2 |
| GH2 | 15 | 13 | 6 | 5 | 3 | 8 | 7 | 10 | 6 | 8 | 10 | 8 |
| GH3 | 25 | 29 | 16 | 16 | 12 | 24 | 23 | 20 | 17 | 32 | 21 | 19 |
| GH4 | 1 | 1 | 0 | 0 | 0 | 0 | 0 | 0 | 0 | 0 | 0 | 0 |
| GH5 | 13 | 12 | 9 | 7 | 6 | 11 | 10 | 16 | 8 | 19 | 12 | 13 |
| GH6 | 1 | 1 | 0 | 1 | 1 | 1 | 1 | 1 | 1 | 0 | 0 | 2 |
| GH7 | 2 | 2 | 1 | 1 | 3 | 3 | 3 | 2 | 1 | 2 | 1 | 6 |
| GH9 | 0 | 0 | 0 | 0 | 0 | 0 | 0 | 0 | 0 | 0 | 0 | 0 |
| GH10 | 2 | 2 | 1 | 0 | 1 | 3 | 5 | 3 | 1 | 4 | 4 | 4 |
| GH11 | 2 | 3 | 3 | 2 | 3 | 4 | 4 | 2 | 4 | 3 | 2 | 4 |
| GH12 | 3 | 2 | 4 | 3 | 3 | 5 | 5 | 4 | 4 | 4 | 4 | 2 |
| GH13 | 19 | 19 | 10 | 18 | 14 | 15 | 14 | 11 | 17 | 10 | 7 | 8 |
| GH15 | 1 | 2 | 2 | 1 | 4 | 2 | 2 | 1 | 1 | 1 | 1 | 1 |
| GH16 | 13 | 12 | 10 | 9 | 11 | 11 | 10 | 7 | 10 | 27 | 21 | 17 |
| GH17 | 4 | 4 | 4 | 5 | 5 | 4 | 5 | 5 | 5 | 6 | 6 | 6 |
| GH18 | 20 | 11 | 7 | 11 | 12 | 13 | 14 | 12 | 12 | 24 | 14 | 14 |
| GH19 | 0 | 0 | 0 | 0 | 0 | 0 | 0 | 0 | 0 | 0 | 0 | 1 |
| GH20 | 3 | 3 | 3 | 2 | 2 | 3 | 3 | 2 | 3 | 4 | 3 | 3 |
| GH23 | 0 | 0 | 0 | 0 | 0 | 0 | 0 | 0 | 0 | 0 | 0 | 0 |
| GH24 | 2 | 2 | 0 | 0 | 0 | 2 | 2 | 2 | 0 | 1 | 0 | 1 |
| GH25 | 2 | 2 | 0 | 0 | 3 | 1 | 1 | 2 | 0 | 0 | 0 | 0 |
| GH26 | 1 | 1 | 1 | 0 | 0 | 1 | 1 | 0 | 1 | 0 | 0 | 0 |
| GH27 | 4 | 4 | 2 | 2 | 1 | 1 | 2 | 3 | 4 | 2 | 2 | 3 |
| GH28 | 11 | 10 | 19 | 19 | 3 | 20 | 20 | 8 | 21 | 15 | 6 | 3 |
| GH29 | 0 | 0 | 1 | 1 | 0 | 0 | 0 | 1 | 1 | 2 | 1 | 3 |
| GH30 | 1 | 1 | 1 | 1 | 1 | 0 | 0 | 1 | 1 | 3 | 0 | 1 |
| GH31 | 11 | 11 | 7 | 5 | 4 | 7 | 7 | 9 | 5 | 9 | 7 | 6 |
| GH32 | 3 | 3 | 2 | 3 | 1 | 3 | 4 | 6 | 5 | 12 | 5 | 3 |
| GH33 | 4 | 5 | 1 | 1 | 0 | 2 | 2 | 1 | 3 | 1 | 1 | 1 |
| GH35 | 3 | 3 | 4 | 4 | 2 | 7 | 6 | 4 | 5 | 6 | 3 | 0 |
| GH36 | 5 | 5 | 2 | 1 | 3 | 3 | 3 | 4 | 3 | 2 | 3 | 1 |
| GH37 | 1 | 1 | 1 | 2 | 1 | 1 | 1 | 1 | 1 | 2 | 2 | 2 |
| GH38 | 1 | 1 | 1 | 1 | 1 | 1 | 1 | 1 | 1 | 1 | 1 | 2 |
| GH39 | 0 | 0 | 0 | 0 | 1 | 0 | 0 | 2 | 0 | 3 | 1 | 2 |
| GH42 | 1 | 1 | 1 | 0 | 0 | 0 | 0 | 0 | 0 | 0 | 0 | 0 |
| GH43 | 39 | 38 | 24 | 31 | 16 | 32 | 28 | 26 | 25 | 53 | 25 | 28 |
| GH45 | 1 | 1 | 0 | 0 | 0 | 0 | 0 | 0 | 0 | 0 | 1 | 1 |
| GH46 | 0 | 0 | 0 | 0 | 0 | 0 | 0 | 0 | 0 | 0 | 0 | 0 |
| GH47 | 6 | 7 | 5 | 5 | 5 | 6 | 5 | 6 | 5 | 10 | 10 | 9 |
| GH49 | 0 | 0 | 0 | 0 | 0 | 0 | 0 | 0 | 0 | 1 | 0 | 0 |
| GH51 | 2 | 2 | 5 | 3 | 3 | 4 | 3 | 4 | 3 | 3 | 2 | 3 |
| GH53 | 1 | 1 | 1 | 1 | 0 | 2 | 1 | 1 | 2 | 2 | 1 | 1 |
| GH54 | 0 | 0 | 0 | 0 | 0 | 0 | 0 | 0 | 0 | 0 | 0 | 0 |
| GH55 | 4 | 4 | 4 | 7 | 5 | 3 | 3 | 5 | 3 | 7 | 4 | 7 |
| GH62 | 2 | 2 | 1 | 1 | 2 | 3 | 2 | 2 | 1 | 1 | 1 | 1 |
| GH63 | 1 | 1 | 1 | 1 | 1 | 1 | 1 | 1 | 1 | 1 | 1 | 2 |
| GH64 | 0 | 0 | 0 | 0 | 0 | 0 | 0 | 0 | 0 | 8 | 2 | 2 |
| GH65 | 1 | 1 | 1 | 1 | 1 | 1 | 1 | 1 | 1 | 1 | 0 | 1 |
| GH67 | 1 | 1 | 1 | 1 | 1 | 1 | 1 | 2 | 1 | 2 | 1 | 1 |
| GH71 | 4 | 4 | 5 | 7 | 3 | 7 | 4 | 4 | 5 | 2 | 1 | 3 |
| GH72 | 2 | 2 | 3 | 3 | 3 | 3 | 3 | 3 | 3 | 2 | 2 | 4 |
| GH73 | 0 | 0 | 0 | 0 | 0 | 0 | 0 | 0 | 0 | 0 | 0 | 0 |
| GH74 | 1 | 1 | 0 | 0 | 0 | 0 | 0 | 0 | 0 | 1 | 1 | 0 |
| GH75 | 2 | 2 | 2 | 2 | 4 | 3 | 3 | 2 | 2 | 2 | 1 | 1 |
| GH76 | 7 | 7 | 9 | 11 | 8 | 11 | 11 | 10 | 11 | 12 | 8 | 8 |
| GH78 | 10 | 9 | 6 | 5 | 0 | 12 | 9 | 4 | 8 | 19 | 7 | 4 |
| GH79 | 2 | 2 | 2 | 2 | 0 | 5 | 4 | 2 | 3 | 6 | 0 | 3 |
| GH81 | 1 | 1 | 1 | 1 | 1 | 1 | 1 | 1 | 1 | 1 | 1 | 2 |
| GH84 | 1 | 1 | 0 | 0 | 0 | 0 | 0 | 0 | 0 | 0 | 0 | 0 |
| GH85 | 0 | 0 | 0 | 0 | 0 | 0 | 0 | 0 | 0 | 0 | 0 | 0 |
| GH88 | 4 | 4 | 1 | 0 | 0 | 3 | 3 | 0 | 1 | 3 | 1 | 1 |
| GH89 | 1 | 1 | 0 | 0 | 1 | 1 | 1 | 0 | 0 | 0 | 0 | 0 |
| GH92 | 5 | 5 | 4 | 4 | 3 | 6 | 6 | 6 | 5 | 0 | 0 | 6 |
| GH93 | 2 | 2 | 2 | 1 | 1 | 3 | 3 | 3 | 0 | 5 | 2 | 1 |
| GH94 | 0 | 0 | 0 | 0 | 0 | 0 | 0 | 0 | 0 | 0 | 0 | 1 |
| GH95 | 2 | 2 | 3 | 2 | 1 | 4 | 3 | 4 | 2 | 2 | 2 | 2 |
| GH97 | 1 | 1 | 0 | 0 | 0 | 0 | 0 | 1 | 0 | 1 | 1 | 0 |
| GH105 | 4 | 4 | 2 | 2 | 3 | 4 | 4 | 2 | 2 | 4 | 3 | 3 |
| GH106 | 0 | 0 | 0 | 0 | 0 | 0 | 0 | 0 | 0 | 0 | 0 | 0 |
| GH109 | 1 | 0 | 0 | 0 | 0 | 0 | 0 | 0 | 0 | 0 | 1 | 0 |
| GH114 | 2 | 2 | 2 | 2 | 1 | 1 | 1 | 1 | 2 | 3 | 2 | 1 |
| GH115 | 4 | 3 | 0 | 0 | 1 | 3 | 2 | 2 | 0 | 3 | 2 | 3 |
| GH125 | 1 | 1 | 1 | 1 | 1 | 1 | 1 | 1 | 1 | 4 | 3 | 3 |
| GH127 | 1 | 1 | 0 | 0 | 1 | 0 | 0 | 1 | 0 | 2 | 1 | 0 |
| GH128 | 3 | 2 | 2 | 2 | 1 | 1 | 1 | 1 | 2 | 4 | 4 | 4 |
| GH130 | 0 | 0 | 0 | 0 | 0 | 0 | 0 | 0 | 0 | 0 | 0 | 0 |
| GH131 | 1 | 1 | 1 | 0 | 1 | 3 | 1 | 1 | 1 | 0 | 1 | 5 |
| GH132 | 2 | 2 | 2 | 1 | 2 | 2 | 2 | 2 | 3 | 2 | 2 | 2 |
| GH134 | 2 | 2 | 1 | 0 | 2 | 3 | 2 | 2 | 0 | 1 | 0 | 0 |
| GH135 | 3 | 2 | 4 | 4 | 2 | 4 | 4 | 1 | 5 | 0 | 0 | 1 |
| GH0 | 5 | 5 | 4 | 4 | 1 | 5 | 3 | 1 | 4 | 6 | 2 | 2 |
| GH0_ | 0 | 0 | 0 | 0 | 0 | 0 | 0 | 0 | 0 | 1 | 0 | 0 |
|  |  |  |  |  |  |  |  |  |  |  |  |  |
| GH2;CBM35 | 1 | 0 | 0 | 0 | 0 | 0 | 0 | 0 | 0 | 0 | 0 | 0 |
| GH5;CBM1 | 0 | 0 | 3 | 2 | 3 | 3 | 1 | 2 | 2 | 2 | 2 | 1 |
| GH5;CBM2;CBM63 | 1 | 1 | 0 | 0 | 0 | 0 | 0 | 0 | 0 | 1 | 0 | 0 |
| GH5_;CBM63 | 0 | 0 | 0 | 0 | 1 | 0 | 0 | 0 | 0 | 1 | 2 | 1 |
| GH6;CBM1 | 0 | 0 | 1 | 0 | 1 | 0 | 0 | 1 | 1 | 1 | 1 | 1 |
| GH7;CBM1 | 1 | 1 | 1 | 1 | 1 | 0 | 0 | 2 | 1 | 1 | 1 | 0 |
| GH10_CBM1 | 0 | 0 | 1 | 0 | 1 | 1 | 0 | 1 | 0 | 2 | 1 | 2 |
| GH11;CBM1 | 0 | 0 | 0 | 0 | 0 | 0 | 0 | 0 | 0 | 0 | 0 | 0 |
| GH12;CBM1 | 0 | 0 | 0 | 0 | 0 | 0 | 0 | 0 | 0 | 0 | 0 | 1 |
| GH13;GT5 | 0 | 0 | 5 | 0 | 0 | 0 | 0 | 0 | 0 | 0 | 0 | 0 |
| GH13;CBM20 | 1 | 1 | 1 | 1 | 2 | 0 | 0 | 2 | 0 | 0 | 0 | 1 |
| GH13;CBM48 | 1 | 1 | 1 | 1 | 1 | 0 | 1 | 1 | 1 | 1 | 1 | 1 |
| GH15;CBM20 | 1 | 1 | 1 | 1 | 2 | 1 | 1 | 1 | 1 | 2 | 2 | 1 |
| GH16;CBM1 | 0 | 0 | 0 | 0 | 0 | 0 | 0 | 0 | 0 | 0 | 0 | 0 |
| GH16;CBM13 | 0 | 0 | 0 | 0 | 0 | 0 | 0 | 0 | 0 | 0 | 0 | 0 |
| GH16;CBM18 | 1 | 1 | 2 | 2 | 1 | 1 | 1 | 1 | 2 | 1 | 1 | 1 |
| GH16;CBM18;CBM18 | 0 | 0 | 0 | 0 | 0 | 0 | 0 | 0 | 0 | 0 | 0 | 2 |
| GH16;CBM6 | 0 | 0 | 0 | 0 | 0 | 0 | 0 | 0 | 0 | 0 | 0 | 0 |
| GH18;CBM1 | 0 | 0 | 0 | 0 | 0 | 0 | 0 | 0 | 0 | 0 | 0 | 0 |
| GH18;CBM18 | 4 | 4 | 6 | 3 | 1 | 1 | 1 | 6 | 4 | 1 | 1 | 5 |
| GH18;CBM18;CBM18 | 5 | 1 | 0 | 3 | 3 | 0 | 0 | 2 | 1 | 4 | 2 | 1 |
| GH18;CBM18;CBM50 | 4 | 3 | 3 | 2 | 0 | 2 | 2 | 3 | 0 | 3 | 1 | 0 |
| GH18;CBM19 | 0 | 0 | 0 | 0 | 1 | 1 | 1 | 0 | 0 | 0 | 0 | 0 |
| GH18;CBM50 | 0 | 0 | 1 | 0 | 0 | 0 | 0 | 0 | 0 | 0 | 1 | 0 |
| GH20_CBM32;CBM32 | 0 | 0 | 0 | 0 | 0 | 0 | 0 | 0 | 0 | 0 | 0 | 0 |
| GH25;CBM50 | 0 | 0 | 0 | 0 | 0 | 0 | 0 | 0 | 0 | 0 | 0 | 0 |
| GH26;CBM35 | 0 | 0 | 0 | 0 | 0 | 0 | 0 | 0 | 0 | 0 | 0 | 0 |
| GH27;CBM13 | 1 | 1 | 1 | 1 | 1 | 1 | 1 | 1 | 1 | 0 | 0 | 0 |
| GH27;CBM35 | 1 | 1 | 1 | 0 | 0 | 1 | 0 | 1 | 0 | 2 | 0 | 0 |
| GH28;CBM1 | 0 | 0 | 0 | 0 | 0 | 0 | 0 | 0 | 0 | 0 | 0 | 0 |
| GH30_CBM1 | 0 | 0 | 0 | 0 | 1 | 0 | 0 | 0 | 0 | 0 | 0 | 0 |
| GH31;DAA35002.1 | 0 | 0 | 0 | 1 | 0 | 1 | 1 | 1 | 1 | 0 | 0 | 0 |
| GH31;GH18 | 0 | 0 | 1 | 0 | 0 | 0 | 0 | 0 | 0 | 0 | 0 | 0 |
| GH32;CBM38 | 0 | 0 | 0 | 0 | 0 | 0 | 0 | 0 | 0 | 4 | 0 | 0 |
| GH35;CBM32 | 1 | 1 | 0 | 0 | 0 | 0 | 0 | 0 | 0 | 0 | 0 | 0 |
| GH43;CBM35 | 0 | 0 | 1 | 0 | 0 | 0 | 0 | 0 | 0 | 0 | 0 | 0 |
| GH43;CBM1 | 1 | 1 | 0 | 0 | 1 | 0 | 0 | 0 | 0 | 0 | 0 | 0 |
| GH43;CBM1;CBM6 | 0 | 0 | 0 | 0 | 0 | 0 | 0 | 0 | 0 | 0 | 0 | 2 |
| GH43;CBM35 | 3 | 3 | 0 | 2 | 1 | 1 | 2 | 1 | 1 | 3 | 2 | 1 |
| GH43;CBM42 | 0 | 0 | 0 | 0 | 0 | 0 | 0 | 0 | 0 | 0 | 0 | 1 |
| GH43;CBM6 | 1 | 1 | 0 | 0 | 0 | 0 | 0 | 1 | 0 | 1 | 1 | 1 |
| GH43;CBM66 | 0 | 0 | 0 | 0 | 0 | 0 | 0 | 0 | 0 | 0 | 0 | 1 |
| GH45;CBM1 | 0 | 0 | 0 | 0 | 0 | 0 | 0 | 0 | 0 | 1 | 0 | 0 |
| GH46;CBM13 | 0 | 0 | 0 | 0 | 0 | 0 | 0 | 0 | 0 | 0 | 0 | 0 |
| GH54;CBM13 | 0 | 0 | 0 | 0 | 0 | 0 | 0 | 0 | 0 | 0 | 0 | 0 |
| GH54;CBM42 | 1 | 1 | 1 | 1 | 1 | 1 | 1 | 1 | 1 | 1 | 1 | 1 |
| GH55;CBM13 | 0 | 0 | 0 | 0 | 0 | 0 | 0 | 0 | 0 | 0 | 0 | 0 |
| GH55;CBM50 | 0 | 0 | 0 | 0 | 0 | 0 | 0 | 0 | 0 | 0 | 0 | 0 |
| GH62;CBM1 | 0 | 0 | 0 | 0 | 0 | 0 | 0 | 1 | 0 | 0 | 0 | 1 |
| GH62;CBM13 | 0 | 0 | 0 | 0 | 0 | 0 | 0 | 0 | 0 | 0 | 0 | 0 |
| GH71;CBM24 | 0 | 0 | 6 | 1 | 0 | 0 | 0 | 1 | 2 | 0 | 0 | 0 |
| GH71;CBM24;CBM24 | 1 | 1 | 0 | 0 | 0 | 2 | 1 | 2 | 0 | 1 | 0 | 0 |
| GH72;CBM43 | 3 | 3 | 2 | 3 | 3 | 4 | 4 | 3 | 4 | 1 | 1 | 1 |
| GH73;CBM50 | 0 | 0 | 0 | 0 | 0 | 0 | 0 | 0 | 0 | 0 | 0 | 1 |
| GH74;CBM1 | 0 | 0 | 1 | 0 | 1 | 0 | 0 | 1 | 1 | 0 | 0 | 0 |
| GH75;CBM13 | 0 | 0 | 0 | 0 | 0 | 0 | 0 | 0 | 0 | 0 | 0 | 0 |
| GH97;CBM13 | 0 | 0 | 0 | 0 | 0 | 0 | 0 | 0 | 0 | 0 | 0 | 0 |
| GH97;CBM51 | 0 | 0 | 0 | 0 | 0 | 0 | 0 | 0 | 0 | 0 | 0 | 0 |
| GH93;CBM66 | 0 | 0 | 0 | 0 | 0 | 0 | 0 | 1 | 0 | 0 | 0 | 0 |
| GH93;CBM13;CBM13 | 1 | 1 | 0 | 0 | 0 | 0 | 0 | 0 | 0 | 0 | 0 | 0 |
| GH131;CBM1 | 0 | 0 | 0 | 0 | 0 | 0 | 0 | 0 | 0 | 0 | 0 | 1 |
| GH133;GH13 | 0 | 0 | 1 | 0 | 0 | 0 | 0 | 0 | 0 | 0 | 0 | 0 |
| GH0_CBM13 | 0 | 0 | 0 | 0 | 0 | 0 | 0 | 0 | 0 | 0 | 1 | 0 |
| GH0_CBM50 | 1 | 1 | 0 | 0 | 0 | 0 | 0 | 2 | 0 | 0 | 0 | 0 |
|  |  |  |  |  |  |  |  |  |  |  |  |  |
|  |  |  |  |  |  |  |  |  |  |  |  |  |
| CE1 | 5 | 5 | 3 | 1 | 4 | 4 | 4 | 3 | 3 | 6 | 3 | 7 |
| CE2 | 0 | 0 | 0 | 0 | 0 | 0 | 0 | 0 | 0 | 1 | 1 | 1 |
| CE3 | 4 | 4 | 0 | 0 | 0 | 3 | 2 | 3 | 0 | 6 | 5 | 6 |
| CE4 | 5 | 5 | 3 | 4 | 3 | 3 | 2 | 3 | 5 | 6 | 7 | 8 |
| CE5 | 5 | 5 | 5 | 6 | 3 | 5 | 3 | 8 | 5 | 11 | 12 | 16 |
| CE6 | 6 | 8 | 15 | 12 | 3 | 9 | 7 | 8 | 14 | 11 | 9 | 4 |
| CE8 | 4 | 3 | 4 | 4 | 3 | 6 | 5 | 2 | 4 | 5 | 4 | 3 |
| CE9 | 1 | 1 | 1 | 3 | 1 | 1 | 1 | 1 | 1 | 1 | 1 | 1 |
| CE12 | 4 | 4 | 3 | 2 | 1 | 4 | 4 | 2 | 2 | 5 | 5 | 2 |
| CE15 | 1 | 1 | 0 | 0 | 1 | 0 | 0 | 1 | 0 | 0 | 0 | 1 |
| CE16 | 5 | 5 | 5 | 3 | 1 | 3 | 3 | 4 | 2 | 5 | 5 | 2 |
| CE0 | 8 | 7 | 4 | 4 | 4 | 5 | 4 | 5 | 5 | 2 | 2 | 1 |
|  |  |  |  |  |  |  |  |  |  |  |  |  |
| CE1;CBM1 | 0 | 0 | 0 | 0 | 1 | 0 | 0 | 1 | 0 | 0 | 0 | 3 |
| CE1;CBM2 | 0 | 0 | 0 | 0 | 0 | 0 | 0 | 0 | 0 | 0 | 0 | 0 |
| CE2;CBM1 | 0 | 0 | 0 | 0 | 0 | 0 | 0 | 0 | 0 | 0 | 0 | 0 |
| CE3;CBM1 | 0 | 0 | 0 | 0 | 0 | 0 | 0 | 0 | 0 | 0 | 0 | 0 |
| CE4;CBM18 | 1 | 1 | 1 | 0 | 0 | 0 | 0 | 1 | 0 | 0 | 1 | 2 |
| CE4;CBM18;CBM18 | 0 | 0 | 0 | 1 | 1 | 1 | 1 | 0 | 1 | 4 | 0 | 1 |
| CE4;CBM50 | 0 | 0 | 0 | 0 | 1 | 0 | 0 | 0 | 0 | 0 | 0 | 0 |
| CE5;CBM1 | 0 | 0 | 0 | 0 | 0 | 0 | 0 | 0 | 0 | 0 | 0 | 0 |
| CE15;CBM1 | 0 | 0 | 0 | 0 | 0 | 0 | 0 | 0 | 0 | 0 | 0 | 0 |
| CE16;CBM1 | 0 | 0 | 0 | 0 | 2 | 0 | 0 | 0 | 0 | 0 | 0 | 0 |
| CE0_CBM3 | 0 | 0 | 0 | 0 | 0 | 1 | 1 | 0 | 0 | 0 | 0 | 0 |
|  |  |  |  |  |  |  |  |  |  |  |  |  |
|  |  |  |  |  |  |  |  |  |  |  |  |  |
| PL1 | 10 | 10 | 6 | 5 | 2 | 12 | 11 | 7 | 6 | 9 | 8 | 2 |
| PL3 | 4 | 4 | 0 | 0 | 1 | 3 | 3 | 3 | 0 | 6 | 7 | 1 |
| PL4 | 4 | 4 | 3 | 2 | 2 | 3 | 2 | 3 | 2 | 3 | 3 | 1 |
| PL7 | 0 | 0 | 1 | 0 | 0 | 1 | 1 | 0 | 0 | 0 | 0 | 0 |
| PL8 | 0 | 0 | 0 | 0 | 0 | 0 | 0 | 0 | 0 | 0 | 0 | 0 |
| PL9 | 1 | 1 | 0 | 0 | 0 | 1 | 1 | 1 | 0 | 2 | 1 | 0 |
| PL11 | 1 | 1 | 0 | 0 | 0 | 0 | 0 | 0 | 0 | 1 | 0 | 0 |
| PL14 | 0 | 0 | 0 | 0 | 0 | 0 | 0 | 0 | 0 | 0 | 0 | 0 |
| PL20 | 0 | 0 | 0 | 0 | 2 | 2 | 2 | 0 | 0 | 0 | 1 | 1 |
|  |  |  |  |  |  |  |  |  |  |  |  |  |
| PL1;CBM1 | 0 | 0 | 0 | 0 | 0 | 0 | 0 | 0 | 0 | 1 | 1 | 0 |
| PL1;CBM35 | 0 | 0 | 0 | 0 | 0 | 0 | 0 | 0 | 0 | 0 | 0 | 0 |
| PL3;CBM1 | 0 | 0 | 0 | 0 | 0 | 0 | 0 | 0 | 0 | 1 | 0 | 0 |
|  |  |  |  |  |  |  |  |  |  |  |  |  |
|  |  |  |  |  |  |  |  |  |  |  |  |  |
| AA1 | 14 | 15 | 11 | 11 | 5 | 13 | 10 | 10 | 16 | 23 | 14 | 16 |
| AA2 | 3 | 3 | 3 | 4 | 3 | 2 | 3 | 3 | 3 | 8 | 5 | 7 |
| AA3 | 33 | 32 | 23 | 30 | 17 | 37 | 33 | 25 | 32 | 32 | 19 | 22 |
| AA4 | 8 | 7 | 1 | 0 | 0 | 2 | 2 | 1 | 2 | 5 | 1 | 1 |
| AA5 | 2 | 2 | 1 | 0 | 1 | 1 | 0 | 0 | 0 | 5 | 6 | 8 |
| AA5_ | 0 | 0 | 0 | 0 | 0 | 0 | 0 | 0 | 0 | 0 | 0 | 2 |
| AA6 | 1 | 1 | 3 | 1 | 1 | 1 | 1 | 1 | 2 | 1 | 1 | 1 |
| AA7 | 40 | 41 | 40 | 32 | 16 | 40 | 36 | 32 | 36 | 48 | 31 | 31 |
| AA8 | 2 | 2 | 2 | 0 | 2 | 1 | 2 | 2 | 2 | 2 | 1 | 4 |
| AA9 | 8 | 8 | 3 | 2 | 5 | 5 | 6 | 7 | 4 | 14 | 10 | 16 |
| AA9_ | 0 | 0 | 0 | 0 | 0 | 0 | 0 | 0 | 0 | 0 | 0 | 2 |
| AA11 | 5 | 5 | 6 | 3 | 5 | 9 | 6 | 8 | 4 | 4 | 5 | 8 |
| AA12 | 0 | 0 | 0 | 0 | 0 | 0 | 0 | 0 | 0 | 3 | 2 | 4 |
| AA13 | 1 | 0 | 0 | 0 | 1 | 1 | 1 | 2 | 0 | 2 | 1 | 0 |
| AA0 | 4 | 5 | 2 | 4 | 1 | 3 | 4 | 2 | 5 | 6 | 4 | 8 |
|  |  |  |  |  |  |  |  |  |  |  |  |  |
| AA3;CBM1 | 0 | 0 | 0 | 0 | 0 | 0 | 0 | 0 | 0 | 1 | 1 | 1 |
| AA5;CBM32 | 1 | 1 | 0 | 1 | 1 | 2 | 2 | 1 | 0 | 3 | 3 | 0 |
| AA3;AA8 | 0 | 0 | 1 | 0 | 0 | 0 | 0 | 0 | 0 | 0 | 0 | 0 |
| AA5;CBM32 | 0 | 0 | 1 | 0 | 0 | 0 | 0 | 0 | 0 | 0 | 0 | 0 |
| AA7;CBM18 | 0 | 0 | 0 | 0 | 0 | 0 | 0 | 0 | 0 | 0 | 0 | 2 |
| AA9;CBM1 | 2 | 2 | 4 | 1 | 2 | 2 | 2 | 5 | 3 | 2 | 2 | 5 |
| AA9;CBM18 | 0 | 0 | 0 | 0 | 0 | 0 | 0 | 0 | 0 | 1 | 1 | 1 |
| AA10_CBM2 | 0 | 0 | 0 | 0 | 0 | 0 | 0 | 0 | 0 | 0 | 0 | 0 |
| AA10_CBM12 | 0 | 0 | 0 | 0 | 0 | 0 | 0 | 0 | 0 | 2 | 0 | 0 |
| AA13;CBM20 | 0 | 1 | 1 | 0 | 1 | 0 | 0 | 0 | 0 | 0 | 0 | 1 |
|  |  |  |  |  |  |  |  |  |  |  |  |  |
|  |  |  |  |  |  |  |  |  |  |  |  |  |
| CBM18 | 0 | 0 | 1 | 0 | 0 | 0 | 0 | 0 | 0 | 0 | 0 | 0 |
| CBM21 | 0 | 0 | 1 | 0 | 0 | 0 | 0 | 0 | 0 | 0 | 0 | 0 |
| CBM50 | 0 | 0 | 7 | 0 | 0 | 0 | 0 | 0 | 0 | 0 | 0 | 0 |
| CBM63 | 0 | 0 | 1 | 0 | 0 | 0 | 0 | 0 | 0 | 0 | 0 | 0 |
|  |  |  |  |  |  |  |  |  |  |  |  |  |
|  |  |  |  |  |  |  |  |  |  |  |  |  |
| GT1 | 8 | 8 | 9 | 9 | 3 | 9 | 7 | 7 | 11 | 20 | 14 | 11 |
| GT2 | 10 | 7 | 8 | 6 | 11 | 9 | 9 | 9 | 8 | 10 | 9 | 8 |
| GT3 | 1 | 1 | 1 | 1 | 1 | 1 | 1 | 1 | 1 | 1 | 1 | 1 |
| GT4 | 7 | 7 | 7 | 7 | 6 | 9 | 9 | 7 | 7 | 7 | 7 | 4 |
| GT8 | 5 | 5 | 5 | 5 | 4 | 3 | 5 | 2 | 4 | 12 | 6 | 2 |
| GT15 | 4 | 3 | 3 | 3 | 3 | 3 | 3 | 3 | 3 | 4 | 4 | 4 |
| GT17 | 0 | 0 | 0 | 0 | 0 | 0 | 0 | 0 | 0 | 2 | 1 | 1 |
| GT18 | 0 | 0 | 0 | 0 | 0 | 0 | 0 | 0 | 0 | 0 | 0 | 0 |
| GT20 | 11 | 9 | 6 | 7 | 7 | 6 | 6 | 6 | 6 | 3 | 3 | 3 |
| GT21 | 1 | 1 | 1 | 1 | 1 | 1 | 1 | 1 | 1 | 1 | 1 | 1 |
| GT22 | 4 | 4 | 4 | 5 | 4 | 4 | 4 | 4 | 3 | 4 | 4 | 4 |
| GT24 | 1 | 1 | 1 | 1 | 1 | 1 | 1 | 1 | 1 | 1 | 1 | 1 |
| GT25 | 3 | 2 | 3 | 5 | 1 | 4 | 4 | 3 | 5 | 0 | 0 | 6 |
| GT31 | 0 | 0 | 0 | 0 | 0 | 1 | 1 | 0 | 0 | 0 | 0 | 0 |
| GT32 | 10 | 10 | 10 | 9 | 7 | 7 | 7 | 9 | 10 | 6 | 4 | 9 |
| GT33 | 1 | 1 | 1 | 1 | 1 | 1 | 1 | 1 | 1 | 1 | 1 | 1 |
| GT34 | 7 | 6 | 2 | 3 | 4 | 2 | 2 | 3 | 3 | 3 | 2 | 2 |
| GT35 | 1 | 1 | 1 | 2 | 1 | 1 | 1 | 1 | 1 | 1 | 1 | 1 |
| GT39 | 3 | 3 | 3 | 3 | 3 | 3 | 3 | 3 | 3 | 3 | 3 | 3 |
| GT41 | 3 | 1 | 1 | 1 | 1 | 1 | 1 | 2 | 1 | 4 | 2 | 2 |
| GT47 | 0 | 0 | 0 | 0 | 0 | 0 | 0 | 0 | 0 | 0 | 0 | 0 |
| GT48 | 1 | 1 | 1 | 1 | 1 | 1 | 1 | 1 | 1 | 1 | 1 | 1 |
| GT50 | 1 | 1 | 1 | 1 | 1 | 1 | 1 | 1 | 1 | 1 | 1 | 1 |
| GT51 | 0 | 0 | 0 | 0 | 0 | 0 | 0 | 0 | 0 | 0 | 0 | 0 |
| GT55 | 0 | 0 | 0 | 0 | 0 | 0 | 0 | 0 | 0 | 0 | 0 | 1 |
| GT57 | 2 | 2 | 2 | 2 | 2 | 2 | 2 | 2 | 2 | 2 | 2 | 2 |
| GT58 | 1 | 1 | 1 | 1 | 1 | 1 | 1 | 1 | 1 | 1 | 1 | 1 |
| GT59 | 1 | 1 | 1 | 1 | 1 | 1 | 1 | 1 | 1 | 1 | 1 | 1 |
| GT62 | 3 | 3 | 3 | 3 | 3 | 3 | 3 | 3 | 3 | 3 | 3 | 3 |
| GT64 | 0 | 0 | 0 | 0 | 0 | 0 | 0 | 0 | 0 | 2 | 2 | 0 |
| GT66 | 1 | 1 | 1 | 1 | 1 | 1 | 1 | 1 | 1 | 1 | 1 | 1 |
| GT69 | 3 | 3 | 4 | 8 | 3 | 4 | 3 | 4 | 6 | 4 | 2 | 3 |
| GT71 | 4 | 4 | 7 | 4 | 4 | 5 | 5 | 4 | 4 | 3 | 4 | 4 |
| GT76 | 1 | 1 | 2 | 2 | 1 | 2 | 1 | 1 | 1 | 1 | 1 | 1 |
| GT90 | 3 | 3 | 3 | 2 | 2 | 4 | 4 | 3 | 3 | 6 | 6 | 6 |
| GT91 | 0 | 0 | 1 | 0 | 0 | 0 | 0 | 1 | 0 | 0 | 0 | 0 |
| GT0 | 1 | 1 | 0 | 0 | 1 | 0 | 0 | 0 | 1 | 2 | 2 | 0 |

**Supplementary Table 7:** Annotation table of the putative and characterized xylanases of the Fsh102, include die names, the contigs, the exons with start and stop, the number of exons and the orientation.

| **Name** | **Name,Type,Minimum,Maximum,Length,#Intervals,Direction** | **Exon_Number, Minimum,Maximum,Direction** | **CDS** | **Genbank number** |
| --- | --- | --- | --- | --- |
| Contig8 Fsh102_84747_GH3 | Fsh102_84747_GH3,gene,111018,113372,2355,1 | exon1,exon,111018,113372,2355 | Contig3, CDS,CDS,111018,113372,2355,1 | BK013308 |
| Fsh102_2 | Fsh102_188581_GH10,gene,66491,67956,1466,1,reverse | \| exon1,exon,67719,67956,238,1,reverse \| \| --- \| \| exon2,exon,67631,67671,41,1,reverse \| \| exon3,exon,67535,67583,49,1,reverse \| \| exon4,exon,67345,67466,122,1,reverse \| \| exon5,exon,67151,67296,146,1,reverse \| \| exon6,exon,67010,67098,89,1,reverse \| \| exon7,exon,66936,66954,19,1,reverse \| \| exon8,exon,66759,66882,124,1,reverse \| \| exon9,exon,66640,66705,66,1,reverse \| \| exon10,exon,66491,66580,90,1,reverse \| | Contig11,CDS,CDS,66491,67956,984,10,reverse | no |
| Xylanase II, Fsh102_1 | Fsh102_63902_GH10,gene,196764,198292,1529,1 | \| exon1,exon,196764,197001,238,1 \| \| --- \| \| exon2,exon,197057,197097,41,1 \| \| exon3,exon,197159,197207,49,1 \| \| exon4,exon,197265,197386,122,1 \| \| exon5,exon,197435,197580,146,1 \| \| exon6,exon,197641,197729,89,1 \| \| exon7,exon,197778,197796,19,1 \| \| exon8,exon,197846,197878,33,1 \| \| exon9,exon,197933,198023,91,1 \| \| exon10,exon,198069,198134,66,1 \| \| exon11,exon,198200,198292,93,1 \| | Contig54,CDS,CDS,196764,198292,987,11 | BK013307 |
| Fsh102_4 | Fsh102_168372_GH11,gene,288971,289698,728,1 | \| exon1,exon,288971,289233,263,1 \| \| --- \| \| exon2,exon,289299,289698,400,1 \| | Contig15,CDS,CDS,288971,289698,663,2 | no |
| Xylanase I, Fsh102_3 | Fsh102_63026_GH11,gene,87419,88075,657,1 | \| exon1,exon,87419,87609,191,1 \| \| --- \| \| exon2,exon,87676,88075,400,1 \| | Contig108,CDS,CDS,87419,88075,591,2 | BK013306 |

**Supplementary Table 8:** Specific activity of purified xylanase I and xylanase II by affinity chromatography. The protein amount (mg), the specific activity (U/mg), the purification quotient (fold) and the yield (%) are listed for each purification step.

| Xylanase I | Total protein  (mg) | | Protein content (mg/mL) | Total activity (U) | Specific activity (U/mg) | Purification  (*fold*) | yield  (%) |
| --- | --- | --- | --- | --- | --- | --- | --- |
| Crude extract | 17.5 ± 1.9 | | 2.1 ± 0.2 | 580.4 ± 35.2 | 33.7 ± 4.8 | 1 ± 0.0 | 100.0 ± 0.0 |
| Flow Through | 13.3 ± 1.6 | | 1.6 ± 0.2 | 158.8 ± 35.2 | 12.0 ± 1.3 | 0.4 ± 0.0 | 27.6 ± 3.5 |
| Wash fraction | 0.9 ± 0.3 | | 0.1 ± 0.0 | 47.5 ± 3 | 59.1 ± 18.2 | 1.7 ± 0.3 | 8.2 ± 0.2 |
| Elution 1 | 1.8 ± 0.2 | | 0.3 ± 0.0 | 315.1 ± 30.7 | 181.1 ± 37.8 | 5.4 ± 0.9 | 54.5 ± 6.1 |
| Elution 2 | 0.1 ± 0.0 | | 0.0 ± 0.2 | 9.3 ± 0.5 | 121.2 ± 9.4 | 3.7 ± 0.4 | 1.6 ± 0.1 |
| Xylanase II | Total protein  (mg) | Protein content (mg/mL) | | Total activity (U) | Specific activity (U/mg) | Purification (fold) | yield  (%) |
| Crude extract | 35.7 ± 4.6 | 4.3 ± 0.6 | | 2029.1 ± 192.9 | 57.1 ± 3.3 | 1 ± 0.0 | 100.0 ± 0.0 |
| Flow Through | 25.2 ± 5.8 | 3.1 ± 0.7 | | 843.4 ± 205 | 33.4 ± 0.5 | 0.6 ± 0.0 | 41.0 ± 6.9 |
| Wash fraction | 4.6 ± 0.6 | 0.6 ± 0.1 | | 366.1 ± 16.9 | 81.4 ± 9.3 | 1.4 ± 0.1 | 18.1 ± 1.2 |
| Elution 1 | 5.2 ± 1.5 | 2.1 ± 0.6 | | 542.8 ± 146.4 | 106.1 ± 15.9 | 1.9 ± 0.3 | 27.2 ± 8.5 |
| Elution 2 | 0.2 ± 0.0 | 0.0 ± 0.0 | | 21.8 ± 3.3 | 121.5 ± 10.9 | 2.1 ± 0.2 | 1.1 ± 0.2 |

**Supplementary Table 9**: Comparison of the temperature and pH stability of the identified xylanases with xylanases described in the literature.

| Enzyme | Reported in | | Activity |
| --- | --- | --- | --- |
| Xylanase I of *Aspergillus sydowii* Fsh102 | | This study | 20% residual activity after 30 min at 50 °C or 70 °C |
| Xylanase II of *Aspergillus sydowii* Fsh102 | | This study | 85% or 20% residual activity after 30 min at 50 °C or 70 °C |
| Xylanase Xyn2 of *Trichoderma reesei* | | ([He et al., 2009](#_ENREF_49)) | 90% residual activity after 30 min at 50 °C, but only 20% at 65-70 °C |
| HoXyl11B of *Hypocrea orientalis* | | ([Li et al., 2018](#_ENREF_64)) | 90% residual activity after 30 min at 50 °C, but only 20% at 65-70 °C |
| GH10 xylanase from *Malbranchea pulchella* | | ([Ribeiro et al., 2014](#_ENREF_84)) | 90% residual activity after almost 1 day at 65 °C |
|  | |  |  |
| Xylanase I and II of *Aspergillus sydowii* Fsh102 | | This study | At least 80% residual activity after 1 day at a pH between 4.8 to 9.0 |
| Xylanase XynC of *Aspergillus kawachii* | | ([Qiu et al., 2016](#_ENREF_81)) | Optimal storage after 3 h incubation at 37 °C found to be at pH 3.8 |
| Different xylanases from *Aspergillus niger* | | ([Krisana et al., 2005](#_ENREF_60); [Fu et al., 2012](#_ENREF_34); [Hmida-Sayari et al., 2012](#_ENREF_52)) | 100% residual activity after 1 h at pH 5 or after 4 h at pH 5 to 10 |
|  | |  |  |

**Supplementary Table 10**: Comparison of the products of the identified xylanases with xylanases described in the literature.

| Enzyme | Reported in | | Observed xylooligosaccharides | Product profile changed during long term incubation |
| --- | --- | --- | --- | --- |
| Xylanase I of *Aspergillus sydowii* Fsh102 (GH11) | | This study | Xylobiose, xylotriose, xylohexose and some higher polymerized xylooligosaccharides | Xylose is appearing |
|  | |  |  |  |
| Xylanase II of *Aspergillus sydowii* Fsh102 (GH10) | | This study | Xylose, xylobiose, xylotriose, xylopentose and some higher polymerized xylooligosaccharides | Xylotriose is disappearing |
| *T. aurantiacus* Xyn10A, *S. lividans* XylA, *A. oryzae* EX, *S. commune* XynB (all 10GH) | | ([Kolenová et al., 2006](#_ENREF_59))* | xylobiose and Aldotetraouronic acid (MeGlcA^3^Xyl_3_) | No information |
|  | |  |  |  |
| *S. commune* XynA, *S. lividans* XynC, *S. thermophile* EX, *T. lanuginosus* EX. | | ([Kolenová et al., 2006](#_ENREF_59))* | Aldopentaouronic acid (MeGlcA^3^Xyl_4_) and xylobiose | No information |
| *S. commune* XynA, *S. lividans* XynC, *S. thermophile* EX, *T. lanuginosus* EX. (all GH11) | |  |  |  |
|  | |  |  |  |
| *Marinifilaceae* Bacterium Strain SPP2 (GH10) | | ([Han et al., 2019](#_ENREF_48)) | Xylobiose, xylotriose, xylotetraose | No information |
| *Aspergillus niger* (GH10) | | ([Takahashi et al., 2013](#_ENREF_101)) | Xylobiose, xylotriose | Xylose is appearing |
|  | |  |  |  |
| *Hypocrea orientalis* (GH11) | | ([Li et al., 2018](#_ENREF_64)) | Xylose, xylobiose, xylotriose | Xylotriose is not disappearing |

* authors used 4-O-methyl-D-glucuronic acid linked XOS

## References

White TJ, Bruns T, Lee S, Taylor J (1990) Amplification and direct sequencing of fungal ribosomal

RNA genes for phylogenetics. In: Innis M, Gelfand D, Sninsky J, White T, editors. PCR Protocols: a

Guide to Methods and Applications. Orlando, Florida: Academic Press. pp. 315-322.

Nielsen H (2017) Predicting Secretory Proteins with SignalP**.** In Kihara, D (ed): *Protein Function Prediction* (Methods in Molecular Biology vol. 1611) pp. 59-73, Springer. doi: [10.1007/978-1-4939-7015-5_6](http://dx.doi.org/10.1007/978-1-4939-7015-5_6)

Brandt SC, Ellinger B, van Nguyen T, Thi QD, van Nguyen G, Baschien C, Yurkov A, Hahnke RL, Schäfer W, Gand M (2018) A unique fungal strain collection from Vietnam characterized for high performance degraders of bioecological important biopolymers and lipids. PLOS ONE 13(8): e0202695. <https://doi.org/10.1371/journal.pone.0202695>

Miller, G.L. (1959). Use of dinitrosalicylic acid reagent for determination of reducing sugar. Anal. Chem. 31. doi: 10.1021/ac60147a030.

Cantarel BL, Coutinho PM, Rancurel C, Bernard T, Lombard V, Henrissat B (2009) The Carbohydrate-Active EnZymes Database (CAZy): An Expert Resource for Glycogenomics. Nucleic Acids Research 37: pp. 233–238.

Lombard V, Ramulu HG, Drula E, Coutinho PM, Henrissat B (2014) The Carbohydrate-Active Enzymes Database (CAZy) in 2013. Nucleic Acids Research 42: pp. 490–495.

Han, ZG, Shang-Guan, F, and Yang, JK (2019). Molecular and Biochemical Characterization of a Bimodular Xylanase From Marinifilaceae Bacterium Strain SPP2. Front. Microbiol. 10. doi: 10.3389/Fmicb.2019.01507.

Kolenová, K, Vrsanska, M, and Biely, P (2006). Mode of action of endo-beta-1,4-xylanases of families 10 and 11 on acidic xylooligosaccharides. J. Biotechnol. 121(3), 338-345. doi: 10.1016/j.jbiotec.2005.08.001.

Takahashi, Y, Kawabata, H, and Murakami, S (2013). Analysis of functional xylanases in xylan degradation by Aspergillus niger E-1 and characterization of the GH family 10 xylanase XynVII. Springerplus 2. doi: 10.1186/2193-1801-2-447.

Li, HL, Wu, HL, Jiang, FJ, Wu, JL, Xue, Y, Gan, LH, et al. (2018). Heterologous Expression and Characterization of an Acidic GH11 Family Xylanase from Hypocrea orientalis. Appl. Biochem. Biotechnol. 184(1), 228-238. doi: 10.1007/s12010-017-2532-2.
